# Synthesis of enantiomerically pure *N*-(2,3-dihydroxypropyl)arylamides via oxidative esterification

**DOI:** 10.3762/bjoc.9.250

**Published:** 2013-10-17

**Authors:** Akula Raghunadh, Satish S More, T Krishna Chaitanya, Yadla Sateesh Kumar, Suresh Babu Meruva, L Vaikunta Rao, U K Syam Kumar

**Affiliations:** 1 Technology Development Centre, Custom Pharmaceutical Services, Dr. Reddy’s Laboratories Ltd, Miyapur, Hyderabad, 500 049, India; 2 Department of Chemistry, GIS, Gitam University, Visakhapatnam, 530 045, India; 3 Integrated Product Development, Innovation Plaza, Dr. Reddy’s Laboratories Ltd, Batchupally, Hyderabad, 500 049, India

**Keywords:** *N*-(2,3-dihydroxypropyl)arylamide, nitrogen heterocyclic carbenes (NHC), oxidative esterification of aromatic aldehyde

## Abstract

A highly efficient synthesis of enantiomerically pure (*S*) and (*R*)-isomers of *N*-(2,3-dihydroxypropyl)arylamides has been developed with good overall yields in a two step process. The key step involves the ring opening of the chiral epoxide with a nitrogen heterocyclic carbene (NHC) and further rearrangement to chiral *N*-(2,3-dihydroxypropyl)arylamides in high yields and enantioselectivity. During the reaction, no erosion in chiral purity was observed.

## Introduction

Chiral structures with three carbons are an integral part of many biologically active compounds including alkaloids, pharmaceuticals and research probes. The development of synthetic routes to these structures is often challenging. Chiral building blocks with three carbon atoms such as glycidol, 1-bromo-2,3-dihydroxypropane and 3-amino-1,2-dihydroxypropane (**1**) are considered powerful tools by synthetic chemists in organic synthesis [1–4].

In the recent past, synthesis of these chiral building blocks has gained significant interest leading to the publication of many reports. The most common methods include (i) reacting a chiral 1,2-propanediol with a leaving group such as a halide or a tosylate ester in the 3-position with base [5–6] or (ii) catalytic oxidations with peroxides and chiral transition metal complexes [7–9]. The oxidative esterification of aldehydes involving oxidation followed by a C–O or C–N bond formation has received significant synthetic interest of late. Various transition metal complexes are employed to facilitate these reactions [10–18]. Herein we describe a highly enantioselective synthesis of (*S*) and (*R*)-*N*-(2,3-dihydroxypropyl)arylamides [19–22] in a two-step process in overall good yields by oxidative esterification of the corresponding aryl aldehydes.

## Results and Discussion

**Scheme 1:**
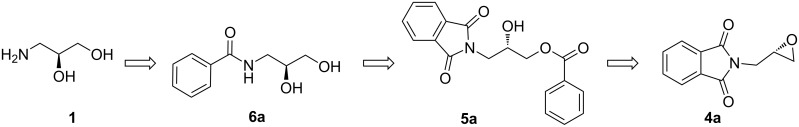
Retro synthetic approach for the construction of *N*-(2,3-dihydroxypropyl)arylamides.

Our strategy for an efficient construction of (*R*) and (*S*)-*N*-(2,3-dihydroxypropyl)benzamide (**6a**) is outlined in Scheme 1. We envisioned that the opening of the phthalimide ring in (*S*)-3-(1,3-dioxoisoindolin-2-yl)-2-hydroxypropyl benzoate (**5a**) would afford the desired benzamide **6a**. The phthalimido-protected chiral hydroxypropyl benzoate **5a** could be synthesized by the reaction of nitrogen heterocyclic carbene, benzaldehyde and phthalimido-epoxide **4a**.

**Scheme 2:**
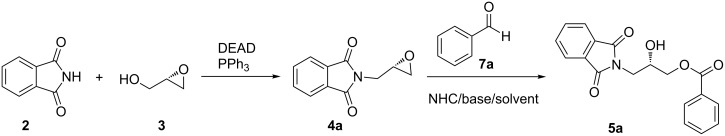
Synthesis of phthalimido-protected chiral hydroxypropyl benzoate.

Phthalimido-epoxide **4a** was synthesized by treating (*S*)-glycidol (**3**) with phthalimide (**2**) under Mitsunobu reaction conditions (Scheme 2). The Mitsunobu reaction yielded the product (*S*)-2-(oxiran-2-ylmethyl)isoindoline-1,3-dione (**4a**) [23–27] in 80% yield and in 99% ee. Then **4a** was converted to hydroxypropyl benzoate **5a** [28–32] by NHC-mediated oxidative esterification of aryl aldehydes (**7a–f**) [33–35]. When the reaction was conducted under nitrogen atmosphere, product formation was not observed. If the reaction was carried out in air, it was found that ester **5a** was obtained as the only product in the presence of NHC. When a diol instead of an epoxide was used as a substrate for the oxidative esterification under these optimized conditions, the expected hydroxypropyl benzoate **5a** was formed in 25–30% yield. Furthermore, additional spots from byproducts were observed on TLC plates.

The opening of the epoxide ring **4a** was attempted with benzaldehyde in the presence of various nitrogen heterocyclic carbenes, such as **9a** to **9k**, with different bases (triethylamine, DBU, and DABCO) and with or without an additive to optimize the reaction conditions (Table 1). When the NHC was used in 0.25 equivalents in THF or THF/butanol (10:1) the major product isolated in the reaction was benzoin, and the desired product (*S*)-3-(1,3-dioxoisoindolin-2-yl)-2-hydroxypropyl benzoate (**5a**) was isolated in only 20% yield. However, when the reaction was performed in NMP at elevated temperatures by using 0.25 equivalent of NHC, the yield of **5a** was significantly improved to 68% along with 10% of benzoin. We did not observe any racemization under these optimized conditions. Higher dilutions of NMP resulted in longer reaction times and lower yields.

**Table 1:** Catalyst screening.^a^

Entry	NHC	Equiv of NHC/base	Solvent (mL)	Temp/time	Yield (%)

1	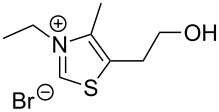 **9a**	0.25/0.25	THF (10)	30 °C/5 h	10
2	**9a**	0.25/0.25	THF:*t*-BuOH (10:1)	30 °C/5 h	20
3	**9a**	0.25/0.25	NMP (10)	80 °C/5 h	30
4	**9a**	0.25/0.25	NMP (7)	80 °C/5 h	35
5	**9a**	0.25/0.4	NMP (3)	80 °C/5 h	68
6	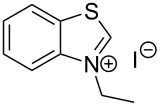 **9b**	0.25/0.25	NMP (3)	80 °C/5 h	35
7	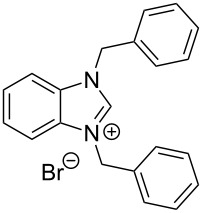 **9c**	0.25/0.25	NMP (3)	80 °C/5 h	63
8	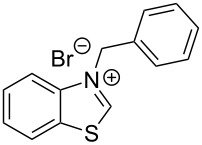 **9d**	0.25/0.25	NMP (3)	80 °C/5 h	42
9	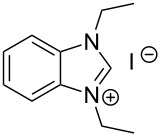 **9e**	0.25/0.25	NMP (3)	80 °C/5 h	50
10	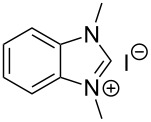 **9f**	0.25/0.25	NMP (3)	80 °C/5 h	45
11	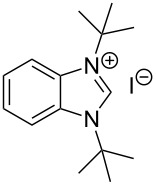 **9g**	0.25/0.25	NMP (3)	80 °C/5 h	60
12	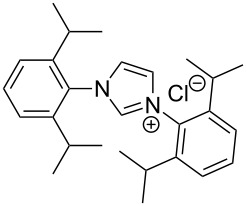 **9h**	0.25/0.4	NMP (3)	80 °C/5 h	67
13	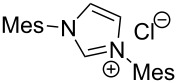 **9i**	0.25/0.4	NMP (3)	80 °C/5 h	69
14	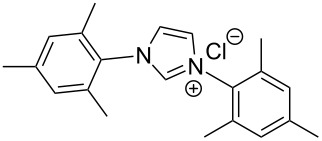 **9j**	0.25/0.4	NMP (3)	80 °C/5 h	64
15	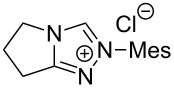 **9k**	0.25/0.4	NMP (3)	80 °C/5 h	61

^a^Optimized reaction conditions: 0.25 equiv of NHC, 0.4 equiv DBU and 3 mL of NMP. Temp: 85–90 °C.

**Scheme 3:**
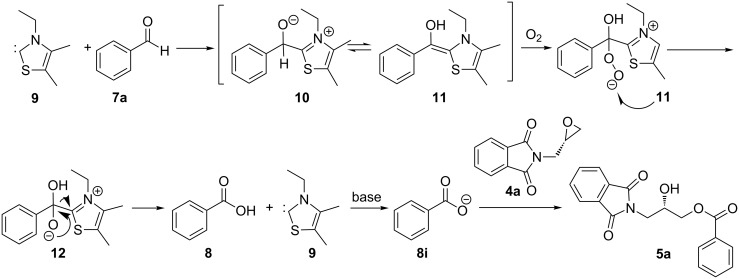
Proposed mechanism of epoxide opening.

A plausible mechanism for the ring opening of epoxide with a nitrogen heterocyclic carbene is presented in Scheme 3. Yadav and co-workers reported the ring opening of epoxide by the Breslow intermediate **11** to provide the corresponding Aldol products [36]. Studer et al. reported the preparation of acids by an oxidation of Breslow intermediates with molecular oxygen [37–39]. The highly activated Breslow intermediate **11** formed by the addition of the NHC to the aldehydes, reacts with dioxygen to form the peroxy-species, which afforded the corresponding hydroxypropyl benzoate. A similar kind of mechanism was also proposed by Ding et al. [40]. To study the mechanism, we conducted a reaction with benzoic acid as a substrate under optimized conditions with or without NHC. Surprisingly, in both cases the formation of the desired product was observed in 40% yield.

The generality of the reaction was proved by synthesizing a variety of optically pure (*R*) and (*S*)-3-(1,3-dioxoisoindolin-2-yl)-2-hydroxypropylarylate derivatives (**5a–h**) in good yields. The representative examples and the reaction conditions are given in Table 2. All the starting epoxides required for this transformation were prepared by using the reported procedures.

**Table 2:** Oxidative esterification of epoxies with NHC.^a^

Entry	Aldehydes	Epoxide	Hydroxypropyl benzoate	Yield (%)	Melting point (°C)

1	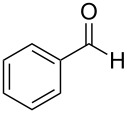 **7a**	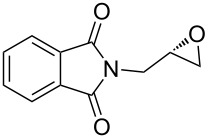 **4a**	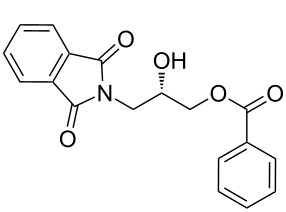 **5a**	68	130–131
2	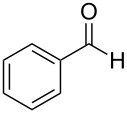 **7a**	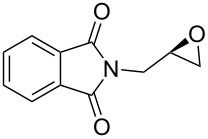 **4b**	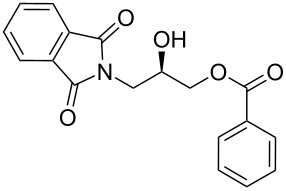 **5b**	68	130–131
3	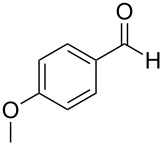 **7b**	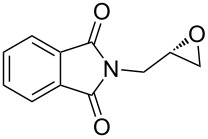 **4a**	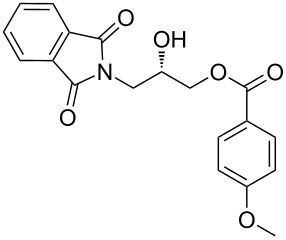 **5c**	63	viscous liquid
4	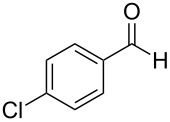 **7c**	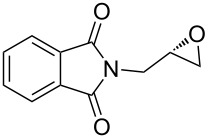 **4a**	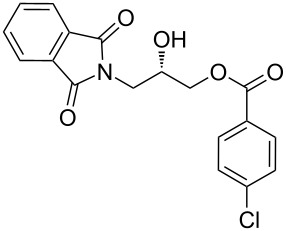 **5d**	65	117–119
5	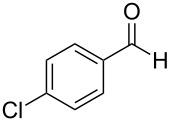 **7c**	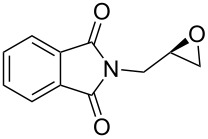 **4b**	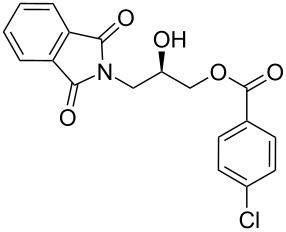 **5e**	65	117–119
6	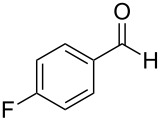 **7d**	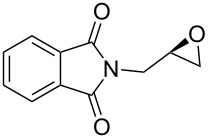 **4b**	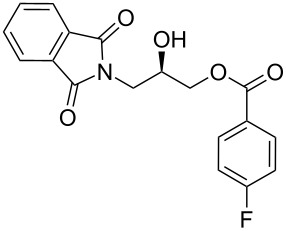 **5f**	64	124–125
7	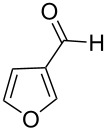 **7e**	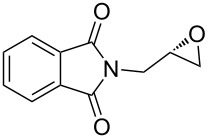 **4a**	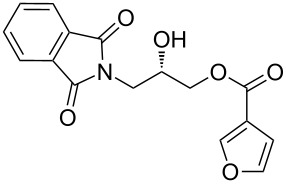 **5g**	60	135–137
8	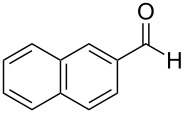 **7f**	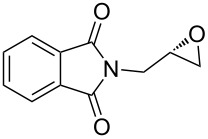 **4a**	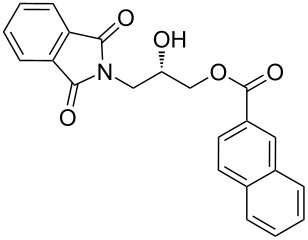 **5h**	59	viscous liquid

^a^All the products were characterized by ^1^H NMR, ^13^C NMR, MS, IR, chiral HPLC and HRMS.

**Scheme 4:**
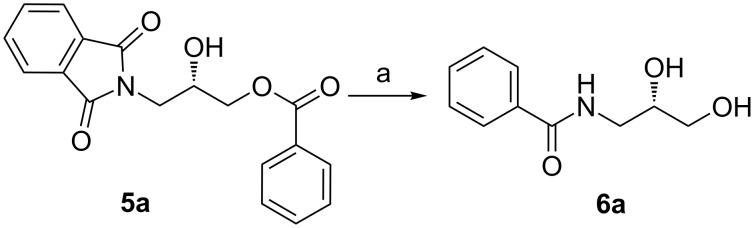
Reagents and conditions: (a) Methylamine, DCM, 30–35 °C, 94%.

After the synthesis of optically pure 3-(1,3-dioxoisoindolin-2-yl)-2-hydroxypropylarylate derivatives (**5a**–**h**), the removal of the phthalimide group was attempted with both methylamine and ammonia solution. During the deprotection of (*S*)-3-(1,3-dioxoisoindolin-2-yl)-2-hydroxypropyl benzylate, we observed the migration of the benzoyl group from the hydroxy group to the amino group (Scheme 4). **6a** was isolated after column chromatographic purifications and showed a chiral HPLC purity of 100% with retention of configuration.

## Conclusion

A highly enantioselective synthesis of (*S*) and (*R*)-isomers of *N*-(2,3-dihydroxypropyl)arylamides was developed with high overall yields. We report the nitrogen heterocyclic carbene catalyzed enantioselective ring opening of chiral epoxide with an aryl or heteroaryl aldehyde by oxidative esterification. This method may prove significant from the perspective of green chemistry. The application of this methodology for the synthesis of several bicyclic frameworks and natural products is under progress, and will be reported in due course.

## Experimental

### General procedure for the synthesis of **5a–h**

Phthalimide epoxide **4a** (10 g, 0.049 mol), NMP (30 mL, 3 equiv), benzaldehyde (5.2 g, 0.049 mol), NHC (3.15 g, 0.0123 mol) and DBU (2.99 g, 0.0197 mol) were mixed in a round bottom flask at 25–30 °C. The reaction mixture was heated to 85–90 °C for 4–5 h. The reaction mixture was then quenched with cold water (300 mL, 10 volume with respect to NMP) and extracted with ethyl acetate (3 × 50 mL). The combined organic layers were washed with water (2 × 50 mL) and brine solution and dried over sodium sulfate. The ethyl acetate layer was concentrated under reduced pressure. The crude product was purified by column chromatography with 20% ethyl acetate in hexane, and the pure product was isolated as pale yellow solid in 68% yield.

**(*S*)-3-(1,3-dioxoisoindolin-2-yl)-2-hydroxypropyl benzoate (5a)**: Yellow solid. Mp 130–131 °C; yield: 10.3 g (68%); IR (KBr): 725, 1025, 1270, 1388, 1425, 1704, 1766, 2994, 3424 cm^−1^; ^1^H NMR (400 MHz, CDCl_3_) δ 3.10 (s, 1H, OH), 3.95–3.97 (m, 2H, CH_2_), 4.39–4.40 (m, 1H, CH), 4.40–4.41 (m, 2H, CH_2_), 7.46 (t, *J* = 8 Hz, 2H, ArH), 7.55 (t, *J* = 7.4 Hz, 1H, ArH), 7.73–7.75 (m, 2H, ArH), 7.86–7.88 (m, 2H, ArH), 8.06 (d, *J* = 7.2 Hz, 2H, ArH); ^13^C NMR (100 MHz, CDCl_3_) δ 41.3, 66.3, 68.4, 123.4, 128.4, 129.6, 129.7, 131.8, 133.1, 134.2, 166.4, 168.7; MS *m*/*z*: 326 [M + 1]; HRMS: calcd for C_18_H_16_NO_5_, 326.1028; found, 326.1031; HPLC: (Chiral PAK-1A (250 × 4.6 mm, column 5.0 u), 1.0 mL/min, 220 nm, *n*-hexane/IPA 80:20, ambient, 5 µL, retention times: 18.75 min, 99.0% ee; [α]_D_^25^ −7.4 (*c* 1.01, methanol).

**(*R*)-3-(1,3-dioxoisoindolin-2-yl)-2-hydroxypropyl benzoate (5b):** Yellow solid: yield: 10.3 g (68%); IR (KBr): 707, 1123, 1271, 1705, 1767, 2944, 3421 cm^−1^; ^1^H NMR (400 MHz, CDCl_3_) δ 3.09 (d, *J* = 6 Hz, 1H, OH), 3.94–3.97 (m, 2H, CH_2_), 4.39–4.40 (m, 1H, CH_2_), 4.40–4.43 (m, 2H, CH_2_), 7.42 (t, *J* = 7.8 Hz, 2H, ArH), 7.55 (t, *J* = 7.4 Hz, 1H, ArH), 7.70–7.77 (m, 2H, ArH), 7.82–7.85 (m, 2H, ArH), 8.06 (d, *J* = 7.0 Hz, 2H, ArH); ^13^C NMR (100 MHz, CDCl_3_) δ 41.2, 66.2, 68.4, 123.4, 128.3, 129.5, 129.6, 131.8, 133.1, 134.1, 166.4, 168.6; MS *m*/*z*: 326 [M + 1]; HRMS: calcd for C_18_H_16_NO_5_, 326.1028; found, 326.1031; HPLC: (Chiral PAK-1A (250 × 4.6 mm, column 5.0 u), 1.0 mL/min, 220 nm, *n*-hexane/IPA 80:20, ambient, 5 µL, retention times: 17.85 min, 98.4% ee; [α]_D_^25^ +7.5 (*c* 0.99, methanol).

**General procedure for the synthesis of N-(2,3-dihydroxypropyl)benzamides (6a and 6b):** To a solution of phthalimido hydroxyl ester (**5a**) (5 g, 0.016 mol) in dichloromethane (5 mL), methylamine (15 mL) was added at 25–30 °C and stirred for 2 h. Sodium hydroxide (0.64 g, 0.016 mol) was added, and the mixture was stirred for another 1–2 h. The reaction mixture was concentrated, and the crude mixture was directly used for column chromatography with 100% ethyl acetate. After column chromatographic purification, the product was isolated as a colorless liquid in 94% yield.

**(*S*)-*N*-(2,3-dihydroxypropyl)benzamide (6a):** Viscous liquid: yield: 2.45 g (94%); IR (KBr): 1027, 1139, 1260, 1515, 2929, 3435 cm^−1^; ^1^H NMR (400 MHz, CDCl_3_) 3.30–3.40 (m, 1H, CH), 3.52–3.59 (m, 3H, CH_2_, CH), 3.81–3.84 (m, 1H, CH), 7.42–7.45 (m, 2H, ArH), 7.50–7.52 (m, 1H, ArH), 7.82–7.83 (m, 2H, ArH); ^13^C NMR (100 MHz, CDCl_3_) δ 43.0, 63.9, 70.4, 127.1, 128.1, 130.9, 134.4, 166.6; MS *m*/*z*: 196 [M + 1]; HRMS: calcd for C_10_H_14_NO_3_, 196.0967; found, 196.0974; HPLC: (Chiral PAK-1A (250 × 4.6 mm, column 5.0 u), 1.0 mL/min, 220 nm, *n*-hexane/IPA 80:20, ambient, 5 µL, retention times: 6.26 min, 100% ee; [α]_D_^25^ −12.44 (*c* 0.75, methanol).

**(*R*)-*N*-(2,3-dihydroxypropyl)benzamide (6b):** Viscous liquid: yield: 2.45 g (94%); IR (KBr): 756, 1116, 1490, 1544, 1637, 2926, 3337 cm^−1^; ^1^H NMR (400 MHz, CD_3_OD) δ 3.30–3.40 (m, 1H), 3.53–3.59 (m, 3H), 3.82–3.85 (m, 1H), 7.43–7.45 (m, 2H), 7.51–7.53 (m, 1H), 7.83–7.84 (m, 2H); ^13^C NMR (100 MHz, CDCl_3_) δ 43.0, 63.9, 70.4, 127.1, 128.1, 130.9, 134.4, and 166.6; HRMS: calcd for C_10_H_14_NO_3_, 196.0967; found, 196.0974; HPLC: (Chiral PAK-1A (250 × 4.6 mm, column 5.0 u), 1.0 mL/min, 220 nm, *n*-hexane/IPA 80:20, ambient, 5 µL, retention times: 6.49 min, 100 % ee; [α]_D_^25^ +12.24 (*c* 0.88, methanol).

## Supporting Information

File 1 Analytical data and NMR, MS and IR spectra.
