# Supplementary material for: Synthesis of enantiomerically pure N-(2,3-dihydroxypropyl)arylamides via oxidative esterification
Source: Beilstein J Org Chem. 2013 Oct 17;9:2129–36. doi: 10.3762/bjoc.9.250 (PMC3817507; doi:10.3762/bjoc.9.250)
Supplement: File 1 — Analytical data and NMR, MS and IR spectra. [file Beilstein_J_Org_Chem-09-2129-s001.pdf]

Supporting Information

for

Synthesis of enantiomerically pure *N*-(2,3-dihydroxypropyl)arylamides via oxidative esterification

Akula Raghunadh*[1], Satish S More[1], T. Krishna Chaitanya[1], Yadla Sateesh Kumar[1], Suresh Babu Meruva[1], L. Vaikunta Rao[2] and U. K. Syam Kumar*[3]

Address: [1]Technology Development Centre, Custom Pharmaceutical Services, Dr. Reddy's Laboratories Ltd, Miyapur, Hyderabad, 500 049, India, [2]Department of Chemistry, GIS, Gitam University, Visakhapatnam, 530 045, India and [3]Integrated Product Development, Innovation Plaza, Dr. Reddy's Laboratories Ltd, Batchupally, Hyderabad, 500 049, India

Email: Akula Raghunadh - raghunadha@drreddys.com; U. K. Syam Kumar - syam_kmr@yahoo.com

Analytical data and NMR, MS and IR spectra

Section A: General information S2

Section B: Analytical………………………………………..…………………….............S3

Section C: ^{1}H, ^{13}C NMR, Mass and IR spectra..S8

Section A: General Information

All reagents were used as received from commercial sources without further purification or prepared as described in the literature. Reactions were stirred using Teflon-coated magnetic stirring bars. Spots on TLC plates were visualized by ultraviolet light or by treatment with a spray of Pancaldi reagent {$(NH_4)_6MoO_4$, $Ce(SO_4)_2$, H_2SO_4, H_2O}. Chromatographic purification of products was carried out by flash column chromatography on silica gel (60–120 mesh). Melting points were determined using an electrothermal melting point apparatus and are uncorrected. Infrared spectra were recorded on a Perkin-Elmer 1650 Fourier transform spectrometer. NMR spectra were measured in $CDCl_3$ or methanol-d_4 (with TMS as internal standard) on a Varian Gemini 400 MHz FT NMR spectrometer magnetic resonance spectrometer. Chemical shifts (δ) are reported in ppm, and coupling constants (*J*) are in Hz. The following abbreviations were used to explain the multiplicities: s = singlet, d = doublet, t = triplet, q = quartet, m = multiplet. Mass spectra were recorded on an HP-5989A quadrapole mass spectrometer

Section B: Analytical

(*S*)-2-(oxiran-2-ylmethyl)isoindoline-1,3-dione (4a):

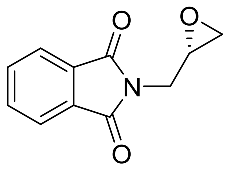

4a

Crystallinesolid,Mp.102-103.5°C, IR (KBr): 3460, 3007,1769, 1713, 1396, 1308, 1049, 961,724. ^{1}H NMR (400 MHz, $CDCl_3$) δ: 2.68-2.70 (m, 1H), 2.80-2.82(m, 1H), 3.22-3.26 (m, 1H), 3.78-3.83 (m, 1H), 3.94-3.99 (m, 1H), 7.75 (dd, *J* = 2.8 Hz, 2H), 7.86 (dd, *J* = 3.2 Hz, 2H); ^{13}C NMR (100 MHz, $CDCl_3$): 39.5, 46.0, 48.9, 123.3, 131.8, 134.0 and 167.8. HPLC: (Chiral PAK-1A (250x4.6mm,column 5.0u), 1.0 ml/min, 220 nm, *n*-hexane/IPA 80:20, Ambient, 5 µL, retention times: 22.35 min, 99.2% ee. Specific Optical Rotation: $[\alpha]_D^{25}$ = 9.0° (c 2.241, Methanol)

(*R*)-2-(oxiran-2-ylmethyl)isoindoline-1,3-dione(4b):

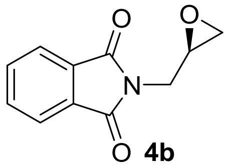

4b

Crystallinesolid, Mp. 101.7-103.5 °C, IR (KBr): 3461, 3006, 1770, 1714, 1398, 1398, 1041, 910, 724, ^{1}H NMR (400 MHz, $CDCl_3$) δ: 2.68-2.70 (m, 1H), 2.80-2.82 (m, 1H), 3.22-3.26 (m, 1H), 3.78-3.80 (m, 1H), 3.94-3.99 (m, 1H), 7.75 (dd, *J* = 2.8 Hz, 2H), 7.86 (dd, *J* = 3.2 Hz, 2H);^{13}C NMR (100 MHz, $CDCl_3$): 39.5, 46.0, 48.9, 123.3, 131.8, 134.0 and 167.8. HPLC: (Chiral PAK-1A (250x4.6mm,column 5.0u), 1.0 ml/min, 220 nm, *n*-hexane/IPA 80:20, Ambient, 5 µL, retention times: 22.35 min, 99.6% ee. Specific Optical Rotation: $[\alpha]_D^{25}$ = −9.1° (c 2.241, Methanol)

(*S*)-3-(1,3-dioxoisoindolin-2-yl)-2-hydroxypropyl 4-methoxybenzoate (5c):

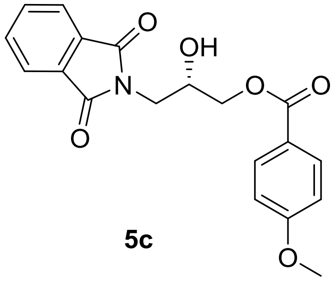

5c

Viscous liquid. yield: (63%). IR (KBr): 756, 1031, 1261, 1395, 1606, 1718, 1774, 3019, cm^{-1}. ^1H NMR (400 MHz, $CDCl_3$) δ: 3.28 (s, 1H, OH), 3.75 (s, 3H, CH_3), 3.84-3.87 (m, 2H, CH_2), 4.21-4.25 (m, 1H, CH), 4.27-4.29 (m, 2H, CH_2), 6.82 (d, *J* = 9.2 Hz, 2H, ArH), 7.60-7.61 (m, 2H, ArH), 7.72-7.73 (m, 2H, ArH), 7.9 0 (d, *J* = 8.8 Hz, 2H, ArH). ^{13}C NMR (100 MHz, $CDCl_3$) = 41.2, 55.3, 66.0, 68.3, 113.6, 121.9, 133.3, 131.6, 131.8, 134.0, 163.4, 166.1, 168.5. MS: m/z = 356 [M + 1], HRMS: Calc. Mass: 356.1570, found 356.1574, Molecular Formula. $C_{19}H_{18}NO_{6.}$, HPLC: (Chiral PAK-1A (250x4.6mm,column 5.0u), 1.0 ml/min, 220 nm, *n*-hexane/IPA 80:20, Ambient, 5 µL, retention times: 22.35 min, 96.6% ee. Specific Optical Rotation: $[\alpha]_D^{25}$ = −10.54° (c 1.04, Methanol)

(*S*)-3-(1,3-dioxoisoindolin-2-yl)-2-hydroxypropyl 4-chlorobenzoate (5d):

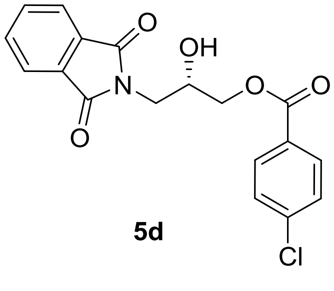

5d

Crystalline solid. yield: (65%). IR (KBr): 722, 760, 1013, 1090, 1271, 1398, 1593, 1699, 1772, 2934, 3474 cm^{-1}. ^1H NMR (400 MHz, $CDCl_3$) δ: 2.9 (s, 1H, OH), 3.9 (d, *J* =5.6 Hz, 2H, CH_2), 4.30-4.32 (m, 1H, CH), 4.39 (d, *J* = 4.8 Hz, 2H, CH_2), 7.42 (d, *J* = 4.8 Hz, 2H,

ArH), 7.74-7.75 (m, 2H, ArH), 7.85-7.86 (m, 2H, ArH), 7.99 (d, J = 8.8 Hz, 2H, ArH). ^{13}C NMR (100 MHz, $CDCl_3$) = 41.3, 66.3, 68.5, 123.5, 128.0, 128.8, 131.1, 131.8, 134.3, 139.7, 165.6, 168.7. MS: m/z = 360 [M + 1], HRMS: Calc. Mass: 360.0639, found : 360.0635, Molecular Formula. $C_{18}H_{15}NO_5Cl$, HPLC: (Chiral PAK-1A (250x4.6mm, column 5.0u), 1.0 ml/min, 220 nm, *n*-hexane: IPA:: 80:20, Ambient, 5 µL, retention times: 23.05 min, 98.2% ee. Specific Optical Rotation: $[\alpha]_D^{25}$ = −0.05° (c 1.00, Methanol)

(*R*)-3-(1,3-dioxoisoindolin-2-yl)-2-hydroxypropyl 4-chlorobenzoate (5e):

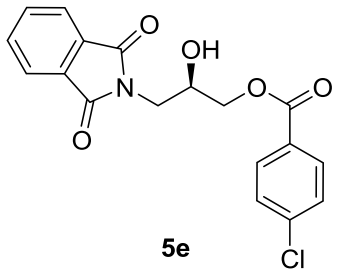

5e

Crystalline solid: yield: (65%). IR (KBr): 721, 755, 1014, 1090, 1272, 1392, 1710, 1731, 1777, 2911, 3503 cm^{-1}. ^{1}H NMR (400 MHz, $CDCl_3$) δ: 2.9 (s, 1H, OH), 3.9 (d, J = 5.6 Hz, 2H, CH_2), 4.30-4.32 (m, 1H, CH), 4.39 (d, J = 4.8 Hz, 2H, CH_2), 7.42 (d, J = 4.8 Hz, 2H, ArH), 7.74-7.75 (m, 2H, ArH), 7.85-7.86 (m, 2H, ArH), 8.0 (d, J = 8.8 Hz, 2H, ArH). ^{13}C NMR (100 MHz, $CDCl_3$) = 41.4, 66.4, 68.5, 123.5, 128.1, 128.7, 131.1, 131.8, 134.2, 139.7, 165.5, 168.6. MS: m/z = 360 [M + 1] HRMS: Calc. Mass: 360.0639, found : 360.0635, Molecular Formula. $C_{18}H_{15}NO_5Cl$, HPLC: (Chiral PAK-1A (250x4.6mm, column 5.0u), 1.0 ml/min, 220 nm, *n*-hexane/IPA 80:20, Ambient,: 5 µL, retention times: 21.72 min, 99% ee Specific Optical Rotation: $[\alpha]_D^{25}$ = +0.05(c 1.00, Methanol).

(*R*)-3-(1,3-dioxoisoindolin-2-yl)-2-hydroxypropyl 4-fluorobenzoate (5f):

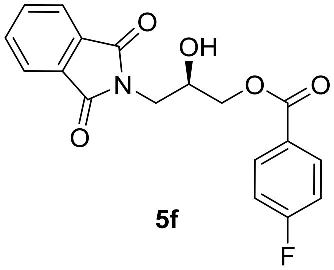

5f

Crystalline solid: yield: (64%). IR (KBr): 951, 1071, 1274, 1604, 1700, 1718, 2997, 3478 cm^{-1}. ^{1}H NMR (400 MHz, $CDCl_3$) δ: 2.96 (d, *J* = 5.2 Hz, 1H, OH), 3.97 (d, *J* = 5.6 Hz, 2H, CH_2), 4.29-4.33 (m, 1H, CH_2), 4.39 (d, *J* = 4.8 Hz, 2H, CH_2), 7.09-7.12 (m, 2H, ArH), 7.73-7.75 (m, 2H, ArH), 7.85-7.87 (m, 2H, ArH), 8.09 (d, *J* = 5.6 Hz, 2H, ArH). ^{13}C NMR (100 MHz, $CDCl_3$) = 41.3, 66.2, 68.5, 115.5, 115.7, 123.5, 131.8, 132.2, 132.3, 134.2, 165.4, 168.7. MS: m/z = 344 [M + 1]. HRMS: Calc. Mass: 344.0934, found : 344.0928. Molecular Formula. $C_{18}H_{15}NO_5F$. HPLC: (Chiral PAK-1A (250x4.6mm,column 5.0u), 1.0 ml/min, 220 nm, *n*-hexane/IPA 80:20, Ambient,: 5 µL, retention times: 22.63 min, 98.1% ee Specific Optical Rotation: $[\alpha]_D^{25}$ = +4.08° (c 1.02, Methanol).

(*S*)-3-(1,3-dioxoisoindolin-2-yl)-2-hydroxypropyl furan-3-carboxylate (5g):

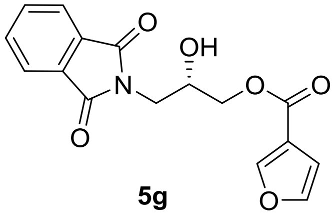

5g

Crystalline solid: yield: (60%). IR (KBr): 761, 1030, 1173, 1309, 1431, 1576, 1706, 1763, 3143, 3384 cm^{-1}. ^{1}H NMR (400 MHz, $CDCl_3$) δ: 3.09 (s, 1H, OH), 3.93-3.94 (m, 2H, CH_2), 4.27-4.29 (m, 1H, CH), 4.33-4.34 (m, 2H, CH_2), 6.75 (d, 1H, ArH), 7.42 (s, 1H, ArH), 7.72-7.73 (m, 2H, ArH), 7.85-7.87 (m, 2H, ArH), 8.09 (d, 1H, ArH). ^{13}C NMR (100 MHz, $CDCl_3$) = 41.2, 65.7, 68.4, 109.7, 118.7, 123.4, 131.7, 134.2, 143.7, 148.1, 162.8,

168.6. MS: m/z = 316 [M + 1], HRMS: Calc. Mass: 316.0821, found :344.0826, Molecular Formula. $C_{16}H_{14}NO_{6.}$ HPLC: (Chiral PAK-1A (250x4.6 mm, column 5.0u), 1.0 ml/min, 220 nm, *n*-hexane/IPA 80:20, Ambient,: 5 µL, retention times: 19.99 min, 99.2% ee Specific Optical Rotation: $[\alpha]_D^{25}$ = −9.49° (c 1.02, Methanol).

(*S*)-3-(1,3-dioxoisoindolin-2-yl)-2-hydroxypropyl 2-naphthoate (5h):

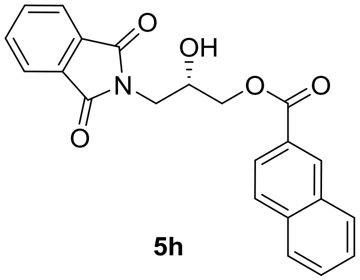

5h

Viscous liquid: yield: (59%). IR (KBr): 780.2, 1096, 1282, 1394, 1467, 1712, 1772, 3467, cm^{-1}. ^{1}H NMR (400 MHz, $CDCl_3$) δ: 3.12 (d, *J* = 4.4 Hz, 1H , OH), 4.02 (d, *J* = 4.4 Hz, 2H, CH_2), 4.37-4.39 (m, 1H, CH), 4.48 (d, *J* = 4.8 Hz, 2H, CH_2), 7.54-7.59 (m , 2H, ArH), 7.70-7.71 (m, 2H, ArH), 7.83-7.87 (m, 4H, ArH), 7.95 (d, *J* = 8 Hz, 1H, ArH), 8.06 (d, *J* = 8.8 Hz, 1H, ArH), 8.61 (s, 1H, ArH). ^{13}C NMR (100 MHz, $CDCl_3$) = 41.1, 66.4, 68.1, 123.2, 125.0, 126.4, 126.7, 127.5, 128.0, 128.2, 129.2, 131.1, 131.6, 132.2, 133.9, 135.4, 166.4, 168.5. MS: m/z = 376 [M + 1], HRMS: Calc. Mass :376.1185, found: 376.1167, Molecular Formula. $C_{22}H_{18}NO_5$

Section C: ^{1}H, ^{13}C NMR, Mass and IR spectra

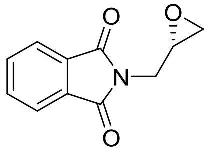

4a

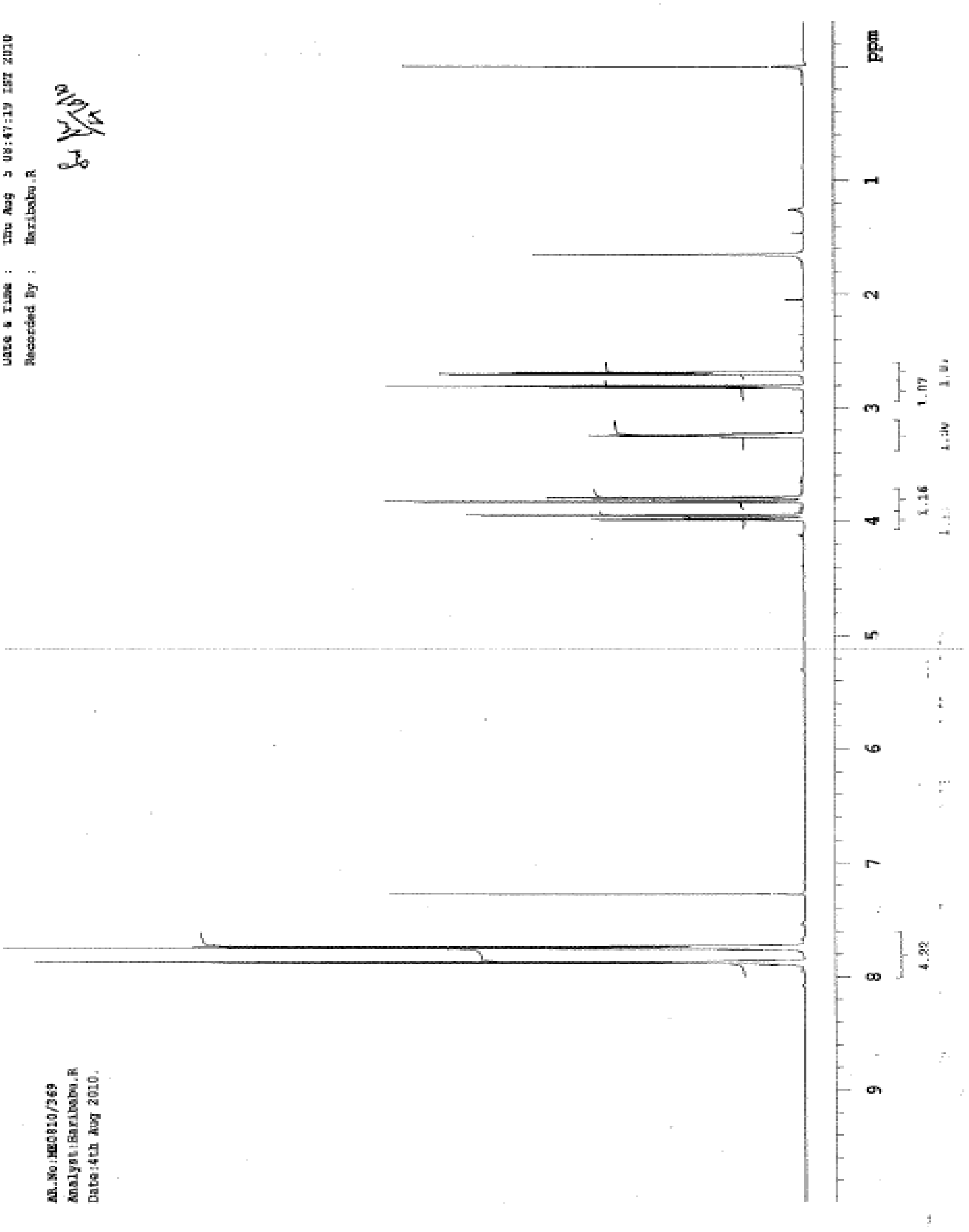

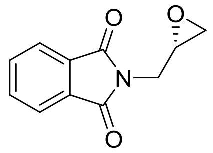

4a

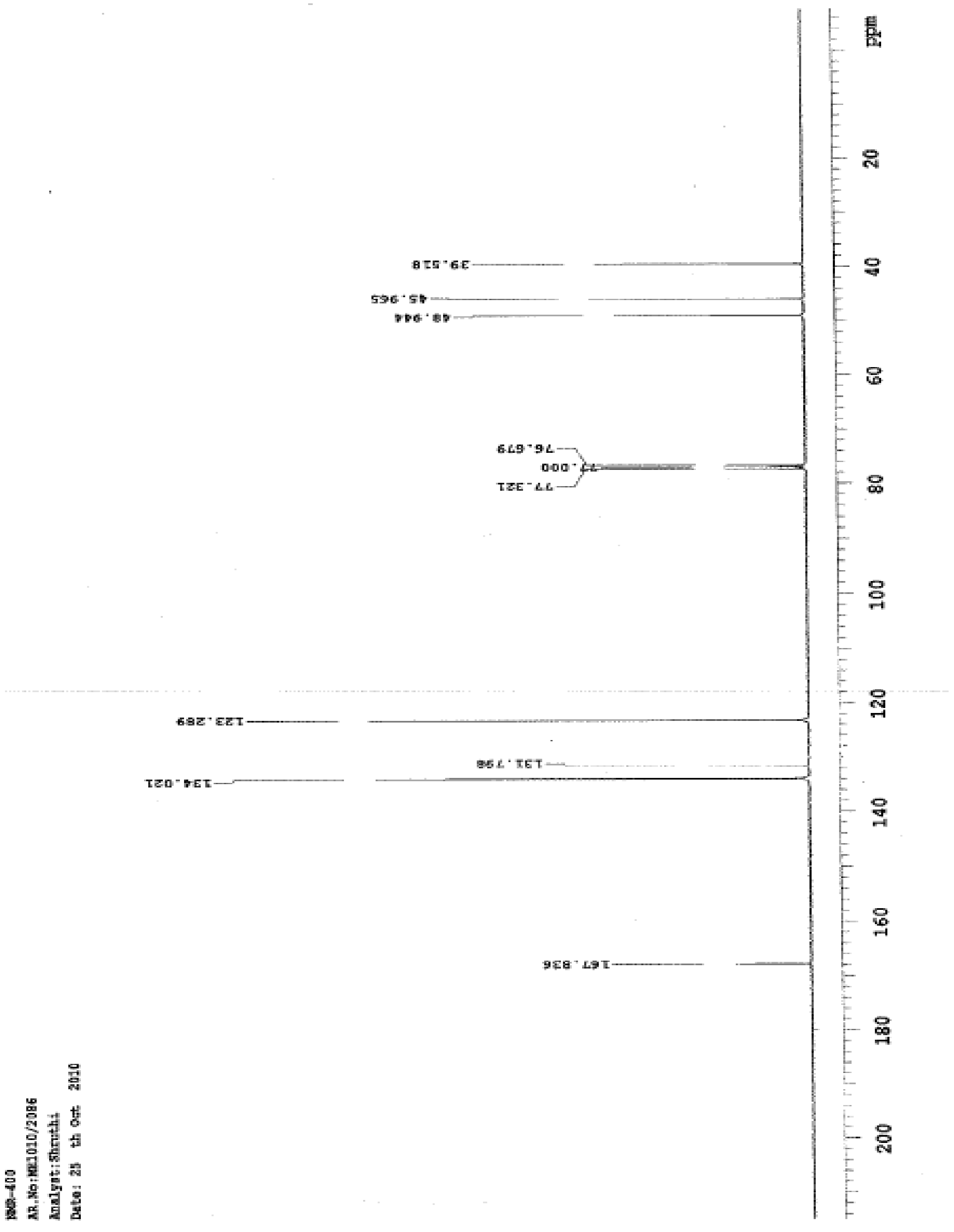

39.518
45.965
48.944
76.679
77.000
77.321
123.289
131.798
134.021
167.836
ppm
20
40
60
80
100
120
140
160
180
200

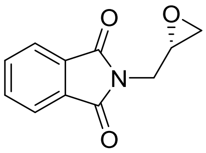

4a

cira2 Report

Reported by User: System   Project Name: HPLC_007_AUG_2010

SAMPLE INFORMATION

Sample Name:	S-EPOXIDE PURE	Acquired By:	System
Sample Type:	Unknown	Date Acquired:	8/12/2010 9:46:56 PM
Vial:	95	Acq. Method Set:	CIRA2
Injection #:	1	Date Processed:	8/13/2010 11:47:44 AM
Injection Volume:	5.00 ul	Processing Methoc	cira2
Run Time:	20.0 Minutes	Channel Name:	WvIn Ch2
Sample Set Name	10081201	Proc. Chnl. Descr.	PDA 220.0 nm

Coloumn: ChiralPAK-IA(250x4.6mm,5.0u)
Mobile Phase: n-Hexane:ipa::80:20
Flow:1.0ml/min,Wave Length:220nm,
Column Temperature:Ambient,Inj Vol:5ul,
Runtime:25min, Dilunt: mp, Conc:0.5mg/mL

Auto-Scaled Chromatogram

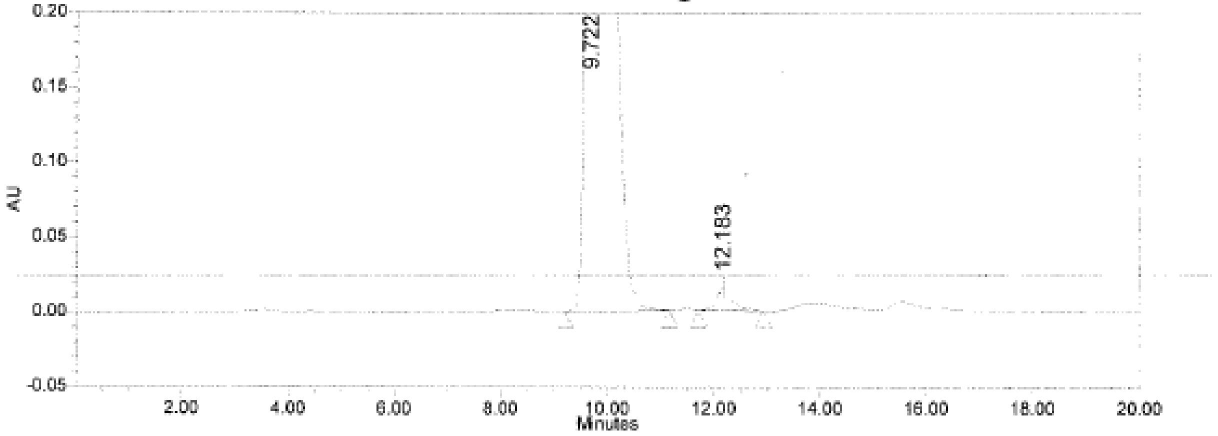

Peak Results

	Name	RT	Area	% Area
1		9.722	47051685	99.32
2		12.183	324028	0.68

~~A.D~~
P.D
13/08/10

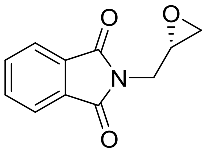

4a

S-Epoxide

[Comment]
Sample name
Comment
User swapna
Workgroup ARND
Division
Company DRL

[Data Information]
Creation Date 8/17/2010 2:40 PM

[Measurement Information]
Instrument Name PL/118/002
Model Name P-2000
Serial No. A035861232
Polarizer Glan-Taylor Prism
Faraday Cell Flint Glass

Accessory PTC-203
Accessory S/N A014261234
Path Length 100 mm

Light Source Na
Monitor wavelength 589 nm
D.I.T. 5 sec
No. of cycle 5
Cycle interval 0 sec
Temp. Monitor Cell
Temp. Corr. Factor None
Aperture(S) 3.0mm
Aperture(L) Auto
Mode Specific O.R.
Path Length 100 mm
Concentration 2.242 w/v
Factor 1

		No.	Sample No.	Mode	Calc. Data	Meas. Data	Monitor(deg)	Temperature(C)	Blank
1	*	1	S-EPOXIDE-1	Specific O.R.	9.3256	0.2091	0.2092	24.83	0.0001
2	*	2	S-EPOXIDE-2	Specific O.R.	9.2409	0.2072	0.2073	24.85	0.0001
3	*	3	S-EPOXIDE-3	Specific O.R.	9.2899	0.2083	0.2084	24.88	0.0001
4	*	4	S-EPOXIDE-4	Specific O.R.	9.2986	0.2085	0.2086	24.91	0.0001
5	*	5	S-EPOXIDE-5	Specific O.R.	9.1873	0.2060	0.2081	24.95	0.0001
6	*	6	Avg.		9.2685				
7		7	S.D		0.0548				
8		8	C.V		0.5908				

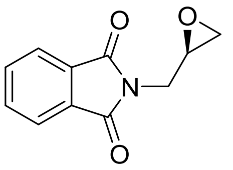

4b

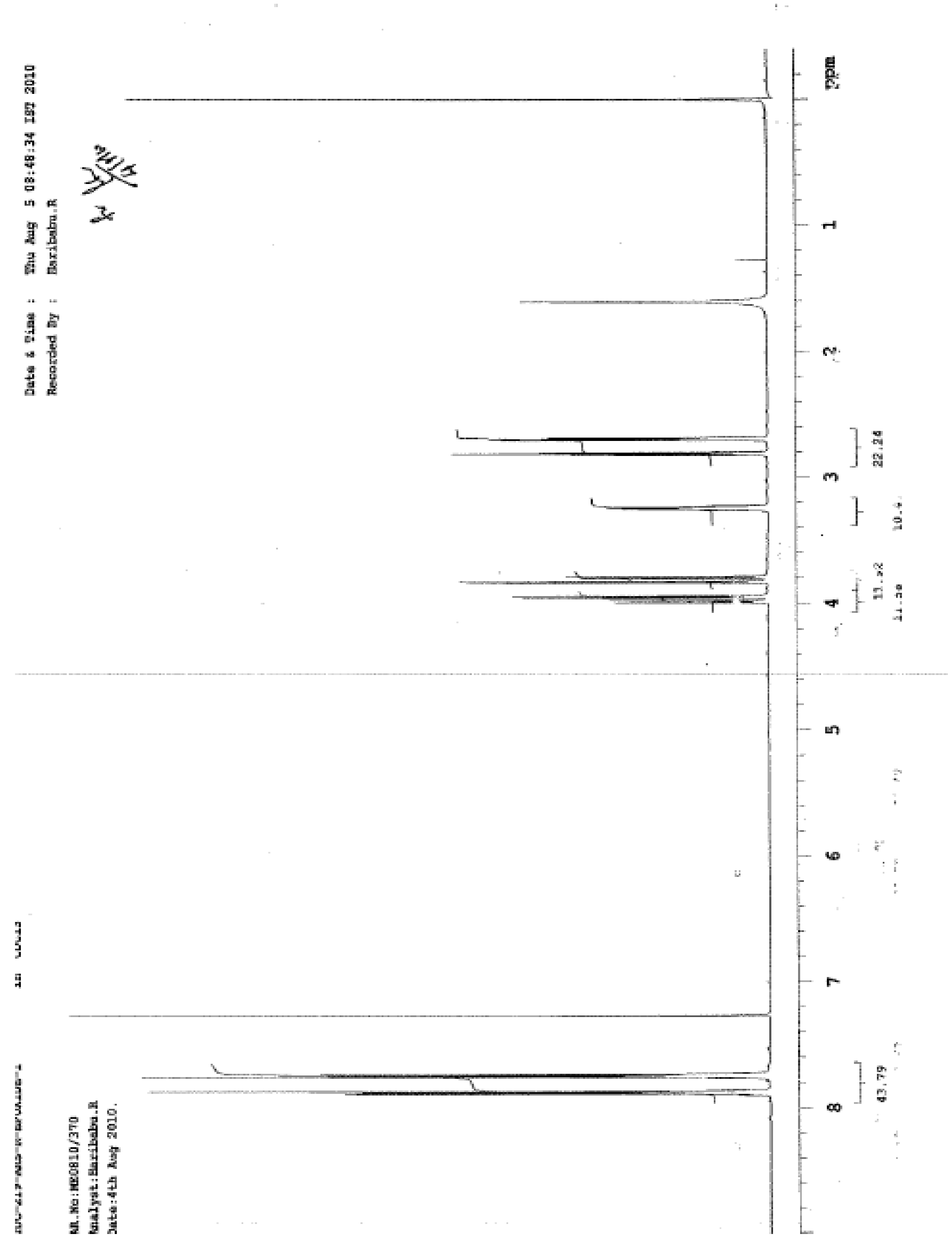
Date & Time : Thu Aug 5 08:48:34 IST 2010
Recorded By : Haribabu.R
AR.No:NK0810/370
Analyst:Haribabu.R
Date:4th Aug 2010.
ppm
22.26
43.79

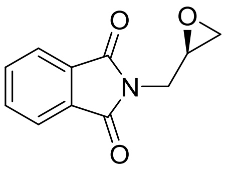
O
N
O
O

4b

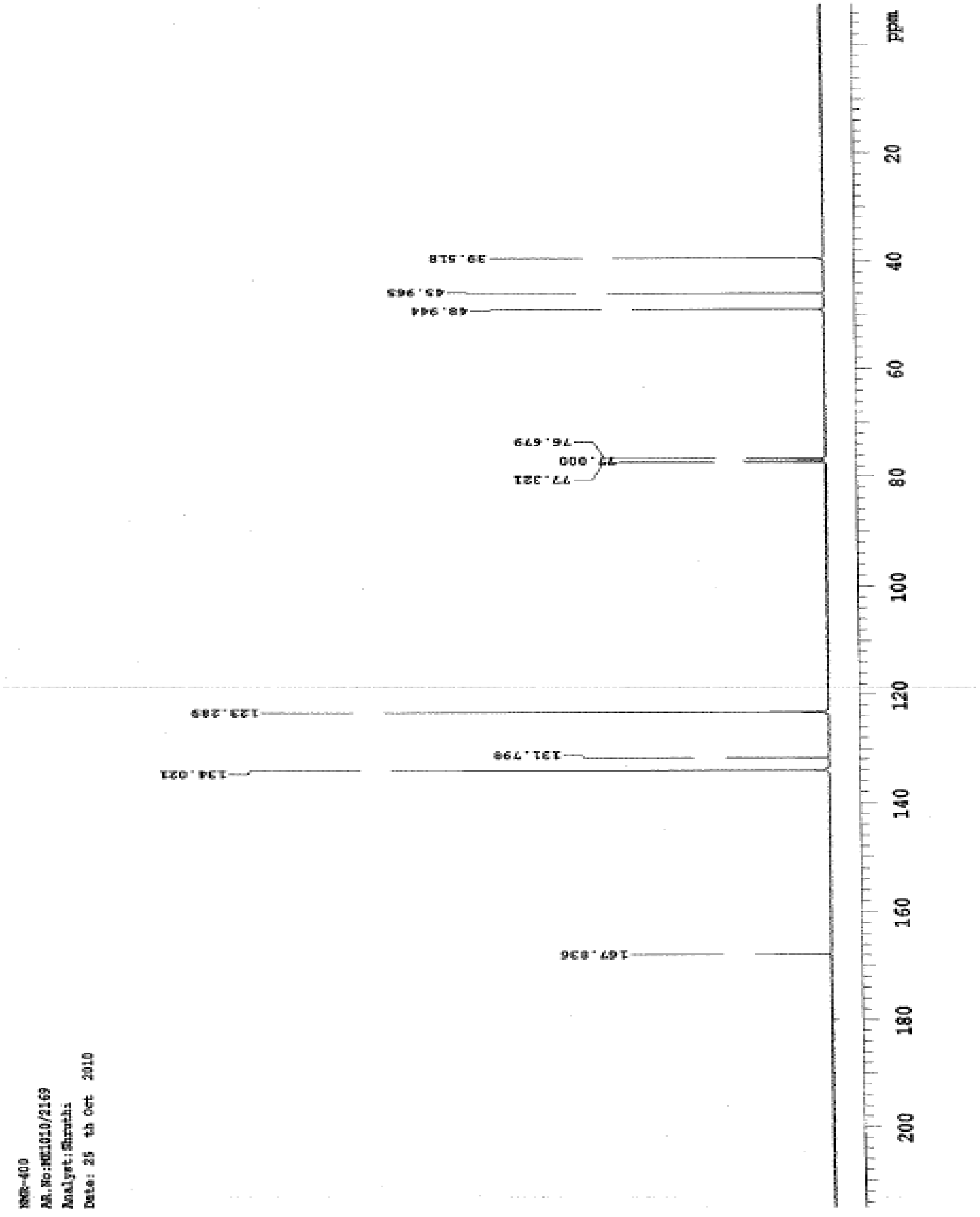
39.518
45.965
48.944
76.679
77.000
77.321
123.289
131.798
134.021
167.836
ppm

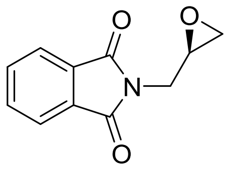

4b

SAMPLE INFORMATION

Sample Name:	CIRA2-EPOXIDE2	Acquired By:	System
Sample Type:	Unknown	Date Acquired:	7/5/2010 2:46:50 PM
Vial:	13	Acq. Method Set:	cira2
Injection #:	1	Date Processed:	7/5/2010 4:50:03 PM
Injection Volume:	5.00 ul	Processing Methoc	CIRA2
Run Time:	30.0 Minutes	Channel Name:	W2996 220.0nm-4.8
Sample Set Name	10070501	Proc. Chnl. Descr.	W2996 PDA 220.0 nm at 4.8

Auto-Scaled Chromatogram

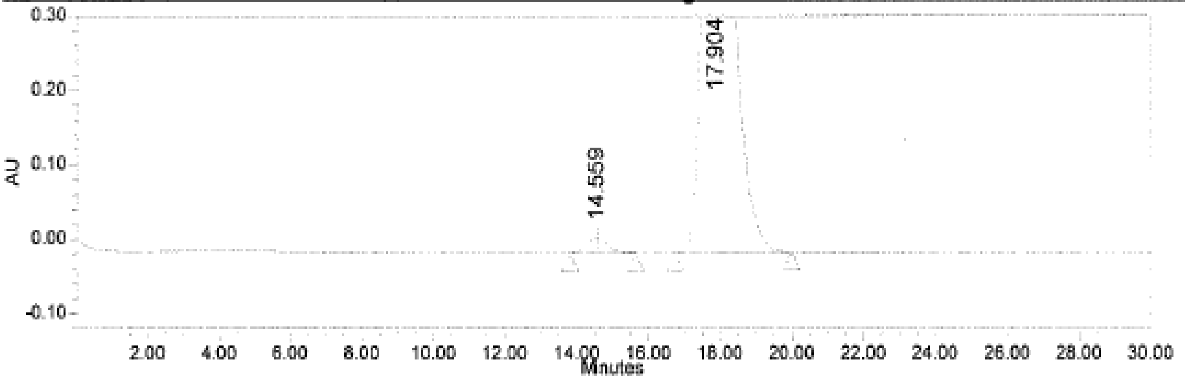

Coloumn: chiral pak IA(250*4.6mm)5µ
Mobile Phase:HEXANE:IA(8:2)
Flow:1.0ml/min, Wave Length:220nm,
Column Temperature:Ambient, Inj Vol:5ul,
Conc: 0.5 mg/ml, Diluent : MP

Peak Results

	Name	RT	Area	% Area
1		14.559	865573	1.92
2		17.904	44284795	98.08

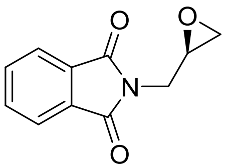

4b

R-Epoxide

[Comment]
Sample name
Comment
User swapna
Workgroup ARND
Division
Company DRL

[Data Information]
Creation Date 8/12/2010 4:50 PM

[Measurement Information]
Instrument Name PL/118/002
Model Name P-2000
Serial No. A035861232
Polarizer Glan-Taylor Prism
Faraday Cell Flint Glass

Accessory PTC-203
Accessory S/N A014261234
Path Length 100 mm

Light Source Na
Monitor wavelength 589 nm
D.I.T. 5 sec
No. of cycle 5
Cycle interval 0 sec
Temp. Monitor Cell
Temp. Corr. Factor None
Aperture(S) 3.0mm
Aperture(L) Auto
Mode Specific O.R.
Path Length 100 mm
Concentration 1.006 w/v
Factor 1

		No.	Sample No.	Mode	Calc. Data	Meas. Data	Monitor(deg)	Temperature(C)	Blank
1	*	1	1-1	Specific O.R.	-7.8330	-0.0788	-0.0966	23.90	-0.0178
2	*	2	1-2	Specific O.R.	-7.7833	-0.0783	-0.0961	23.93	-0.0178
3	*	3	1-3	Specific O.R.	-7.8429	-0.0789	-0.0967	23.95	-0.0178
4	*	4	1-4	Specific O.R.	-7.7833	-0.0783	-0.0961	23.95	-0.0178
5	*	5	1-5	Specific O.R.	-8.3002	-0.0835	-0.1013	23.96	-0.0178
6	*	6	Avg.		-7.9085				
7		7	S.D		0.2207				
8		8	C.V		2.7902				
9	*	9	R EPOXIDE 1a-1	Specific O.R.	-8.8668	-0.0892	-0.1070	23.86	-0.0178
10	*	10	R EPOXIDE 1a-2	Specific O.R.	-9.2346	-0.0929	-0.1107	23.86	-0.0178
11	*	11	R EPOXIDE 1a-3	Specific O.R.	-9.0457	-0.0910	-0.1088	23.86	-0.0178
12	*	12	R EPOXIDE 1a-4	Specific O.R.	-9.0557	-0.0911	-0.1089	23.86	-0.0178

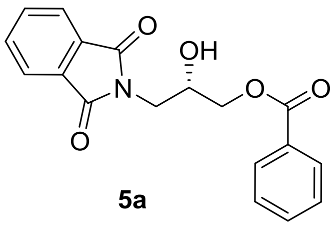
O
OH
N
O
O
O

5a

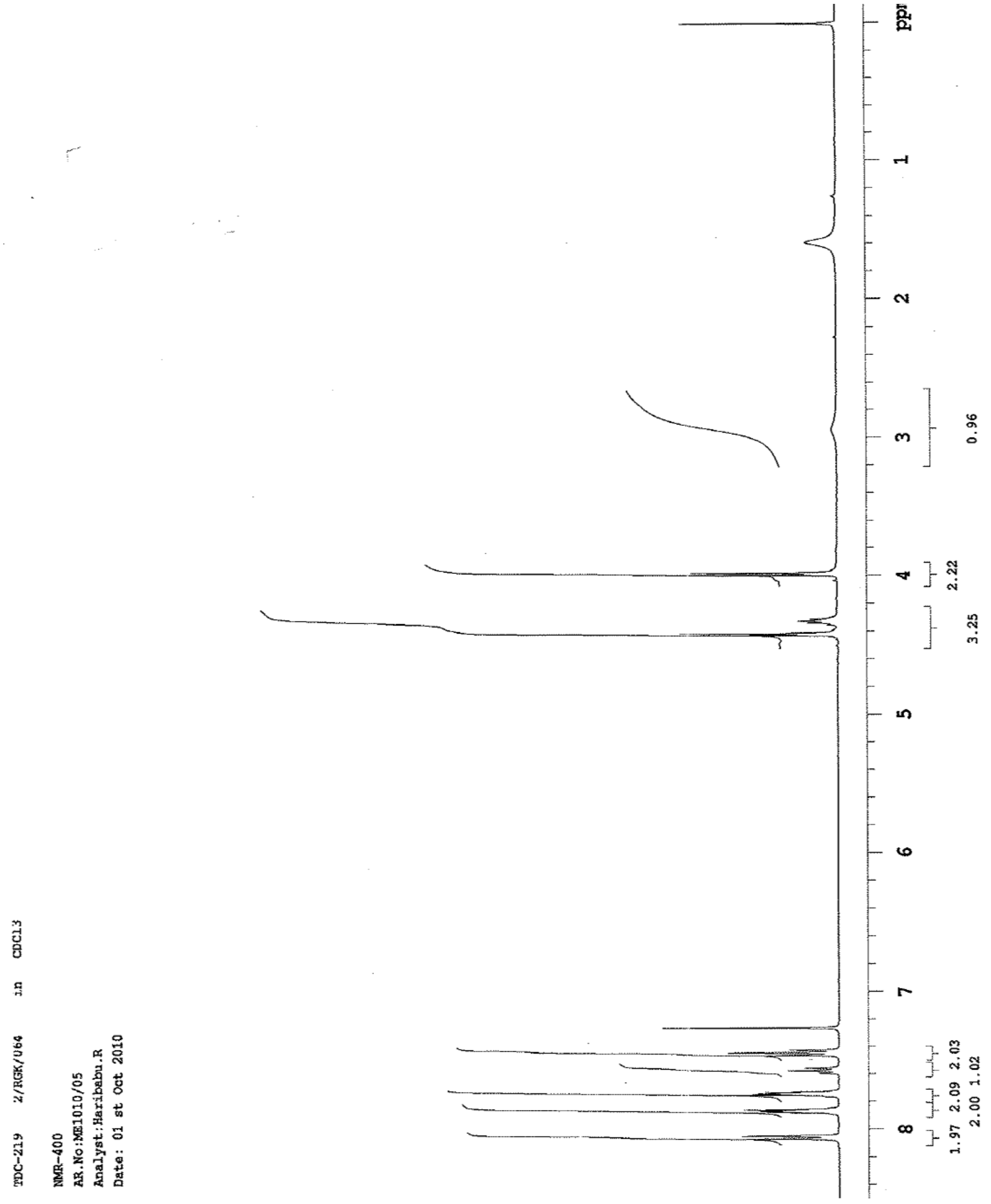
TDC-219
CDCl3
NMR-400
AR.No:ME1010/05
Analyst:Haribabu.R
Date: 01 st Oct 2010
ppm
1
2
3
4
5
6
7
8
0.96
2.22
3.25
1.97 2.09 2.03
2.00 1.02

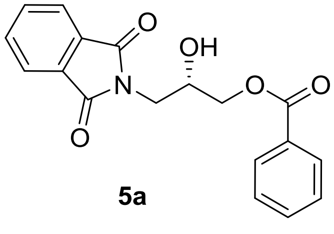
O
OH
N
O
O
O

5a

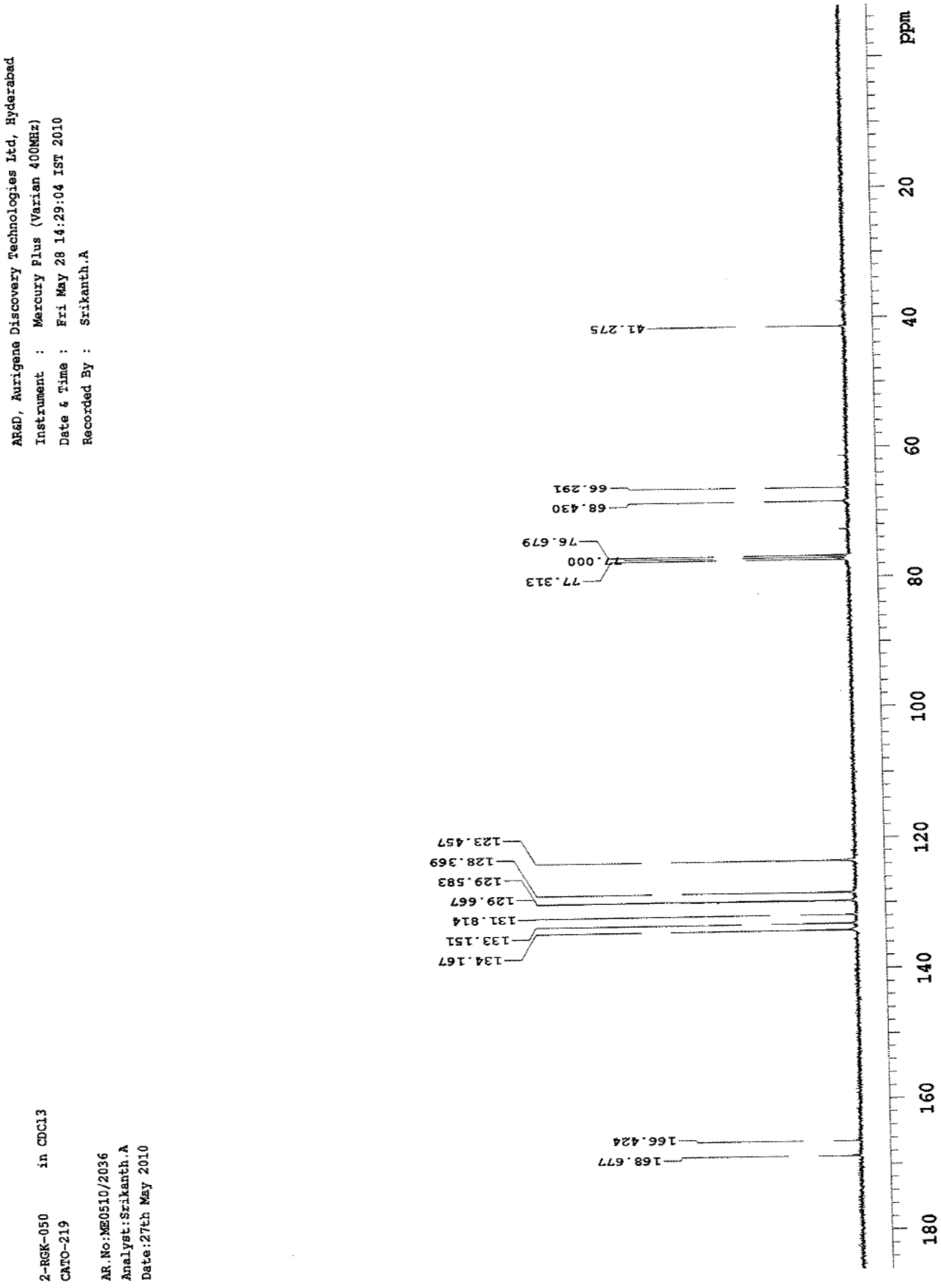
AR&D, Aurigene Discovery Technologies Ltd, Hyderabad
Instrument : Mercury Plus (Varian 400MHz)
Date & Time : Fri May 28 14:29:04 IST 2010
Recorded By : Srikanth.A
2-RGK-050 in CDCl3
CATO-219
AR.No:ME0510/2036
Analyst:Srikanth.A
Date:27th May 2010
41.275
66.291
68.430
76.679
77.000
77.313
123.457
128.369
129.583
129.667
131.814
133.151
134.167
166.424
168.677
20
40
60
80
100
120
140
160
180
ppm

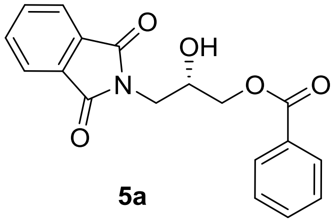

5a

2-RGK-064.p1d

[Comment]
Sample name
Comment
User ramanjaneyulu
Workgroup ARND
Division
Company DRL

[Data Information]
Creation Date 10/1/2010 11:06 AM

[Measurement Information]
Instrument Name PL/118/002
Model Name P-2000
Serial No. A035861232
Polarizer Glan-Taylor Prism
Faraday Cell Flint Glass

Accessory PTC-203
Accessory S/N A014261234
Path Length 100 mm

Light Source Na
Monitor wavelength 589 nm
D.I.T. 5 sec
No. of cycle 5
Cycle interval 0 sec
Temp. Monitor Cell
Temp. Corr. Factor None
Aperture(S) 3.0mm
Aperture(L) Auto
Mode Specific O.R.
Path Length 100 mm
Concentration 1.0123 w/v
Factor 1

		No.	Sample No.	Mode	Calc. Data	Meas. Data	Monitor(deg)	Temperature(C)	Blank
1	*	1	12-RGK-064-1	Specific O.R.	-7.6045	-0.0770	-0.0815	23.63	-0.0045
2	*	2	12-RGK-064-2	Specific O.R.	-7.2587	-0.0735	-0.0780	23.71	-0.0045
3	*	3	12-RGK-064-3	Specific O.R.	-7.5946	-0.0769	-0.0814	23.78	-0.0045
4	*	4	12-RGK-064-4	Specific O.R.	-7.6440	-0.0774	-0.0819	23.83	-0.0045
5	*	5	12-RGK-064-5	Specific O.R.	-7.4464	-0.0754	-0.0799	23.89	-0.0045
6	*	6	Avg.		-7.5096				
7		7	S.D		0.1591				
8		8	C.V		2.1182				

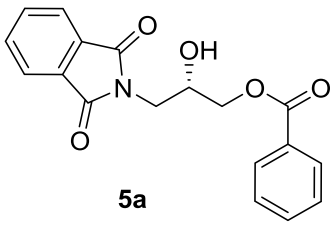

5a

SAMPLE INFORMATION

Sample Name:	S-related aldehyde	Acquired By:	System
Sample Type:	Unknown	Date Acquired:	8/12/2010 7:21:26 PM
Vial:	89	Acq. Method Set:	CIRA2
Injection #:	1	Date Processed:	8/13/2010 11:45:45 AM
Injection Volume:	5.00 ul	Processing Method	cira2
Run Time:	40.0 Minutes	Channel Name:	Wvln Ch2
Sample Set Name	10081201	Proc. Chnl. Descr.	PDA 220.0 nm

Coloumn: ChiralPAK-IA(250x4.6mm,5.0u)
Mobile Phase: n-Hexane:ipa::80:20
Flow:1.0ml/min,Wave Length:220nm,
Column Temperature:Ambient,Inj Vol:5ul,
Runtime:25min, Dilunt: mp, Conc:0.5mg/mL

Auto-Scaled Chromatogram

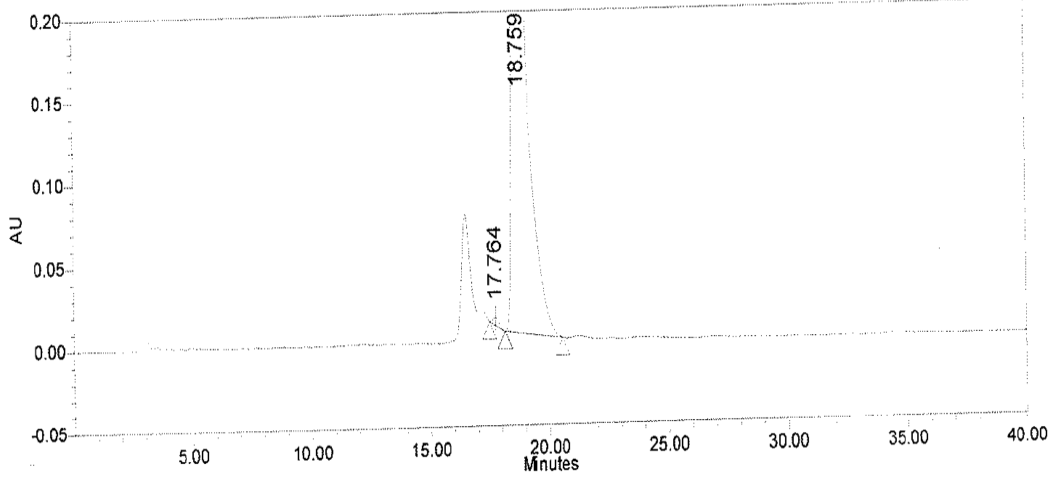

Peak Results

	Name	RT	Area	% Area
1		17.764	84596	0.48
2		18.759	17568524	99.52

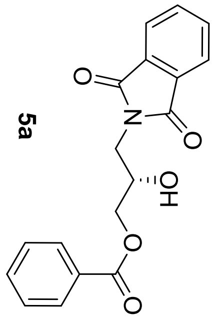

5a

Elemental Composition Report

Page 1

Single Mass Analysis
Tolerance = 5.0 PPM / DBE: min = -1.5, max = 80.0
Element prediction: Off
Number of isotope peaks used for i-FIT = 3

Monoisotopic Mass, Even Electron Ions
112 formula(e) evaluated with 2 results within limits (up to 4 best isotopic matches for each mass)
Elements Used:
C: 0-30 H: 0-30 N: 0-5 O: 0-6
2/RGK/067
UT1211_030 18 (0.339) Cm (18:23)

1: TOF MS ES+
6.48e+003

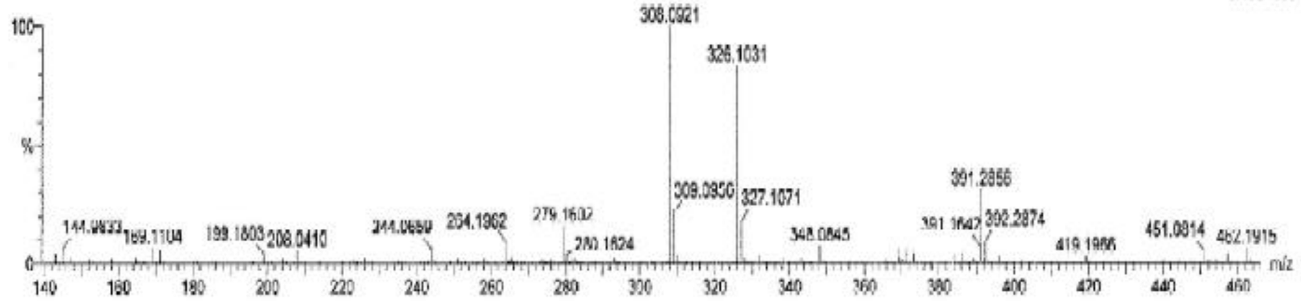

Minimum:				-1.5		
Maximum:		5.0	5.0	80.0		
Mass	Calc. Mass	mDa	PPM	DBE	i-FIT	Formula
326.1031	326.1028	0.3	0.9	11.5	0.9	C18 H16 N O5
	326.1042	-1.1	-3.4	16.5	3.1	C19 H12 N5 O

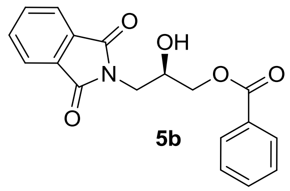
OH
O
N

5b

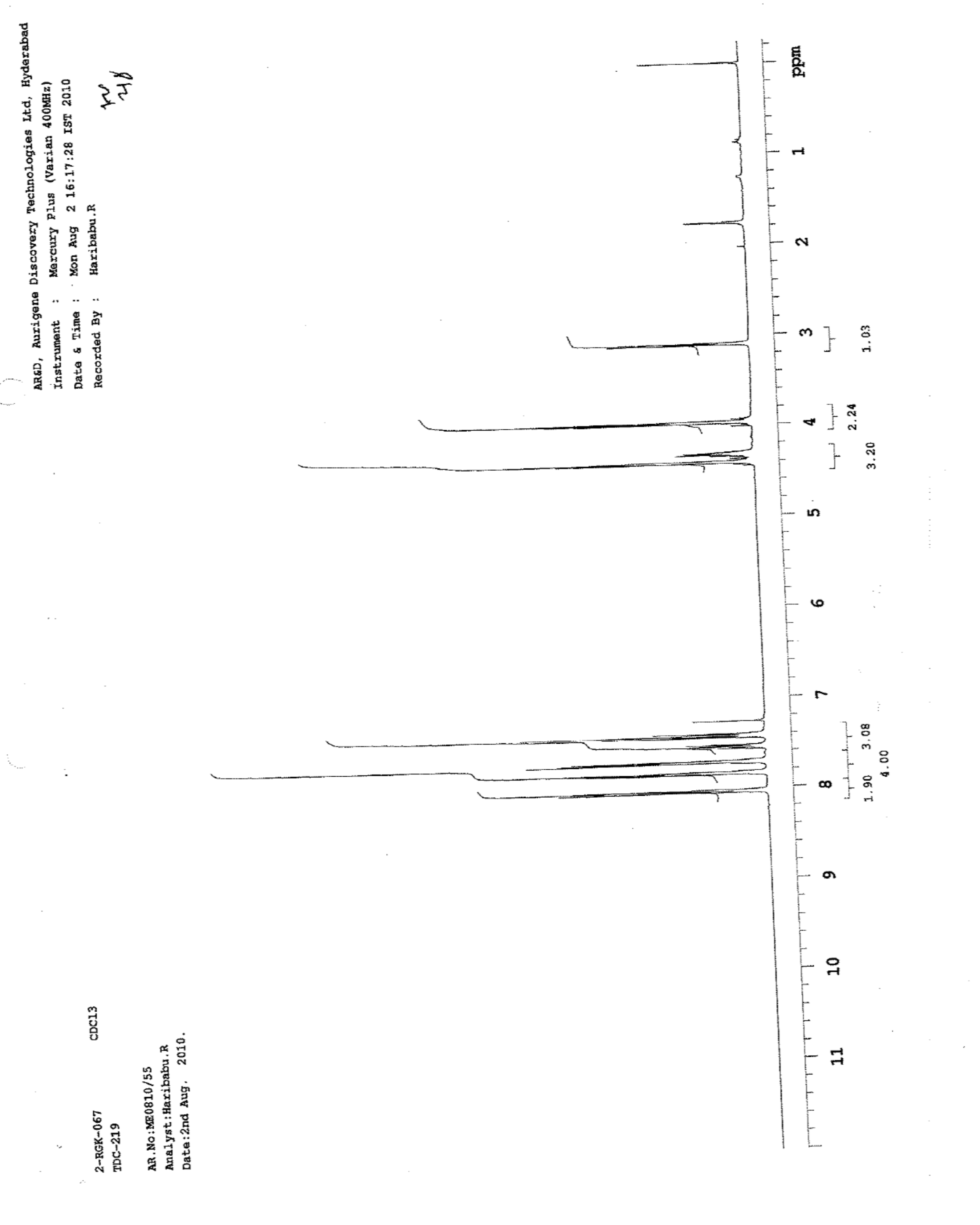
AR&D, Aurigene Discovery Technologies Ltd, Hyderabad
Instrument : Mercury Plus (Varian 400MHz)
Date & Time : Mon Aug 2 16:17:28 IST 2010
Recorded By : Haribabu.R
CDCl3
2-RGK-067
TDC-219
AR.No:ME0810/55
Analyst:Haribabu.R
Date:2nd Aug. 2010.
ppm
1.03
2.24
3.20
3.08
1.90
4.00

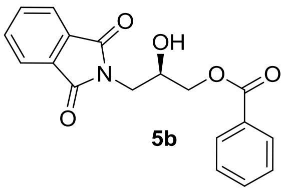
OH
N
O
O
O
O

5b

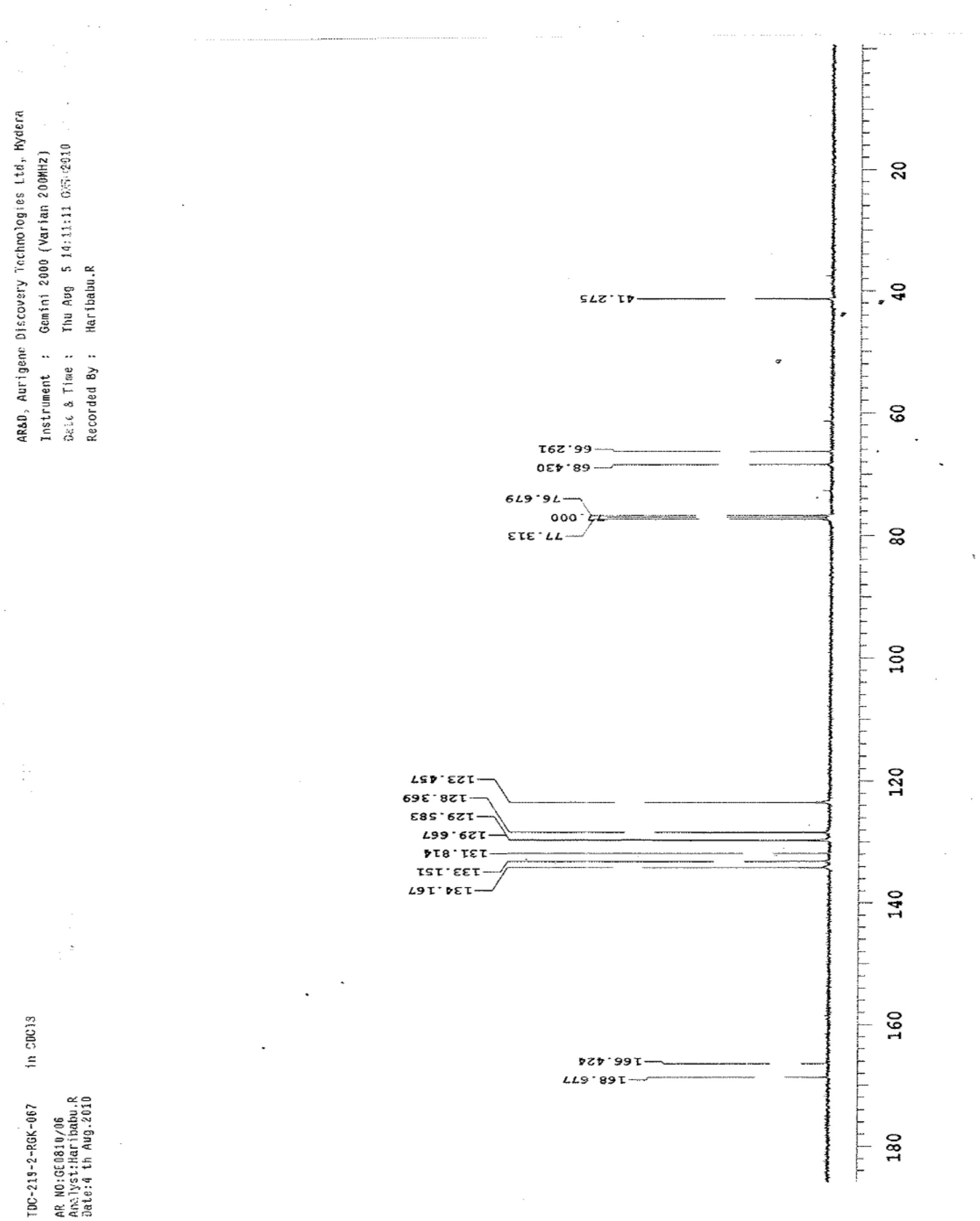
AR&D, Aurigene Discovery Technologies Ltd, Hydera
Instrument : Gemini 2000 (Varian 200MHz)
Recorded By : Haribabu.R
TDC-219-2-RGK-067 in CDCl3
AR NO:GE0810/06
Analyst:Haribabu.R
Date:4 th Aug.2010
41.275
66.291
68.430
76.679
77.000
77.313
123.457
128.369
129.583
129.667
131.814
133.151
134.167
166.424
168.677
20
40
60
80
100
120
140
160
180

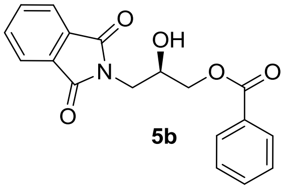

5b

2-RGK-067.p1d

[Comment]
Sample name
Comment
User ramanjaneyulu
Workgroup ARND
Division
Company DRL

[Data Information]
Creation Date 10/1/2010 11:15 AM

[Measurement Information]
Instrument Name PL/118/002
Model Name P-2000
Serial No. A035861232
Polarizer Glan-Taylor Prism
Faraday Cell Flint Glass

Accessory PTC-203
Accessory S/N A014261234
Path Length 100 mm

Light Source Na
Monitor wavelength 589 nm
D.I.T. 5 sec
No. of cycle 5
Cycle interval 0 sec
Temp. Monitor Cell
Temp. Corr. Factor None
Aperture(S) 3.0mm
Aperture(L) Auto
Mode Specific O.R.
Path Length 100 mm
Concentration 0.9912 w/v
Factor 1

		No.	Sample No.	Mode	Calc. Data	Meas. Data	Monitor(deg)	Temperature(C)	Blank
1	*	1	2-RGK-067-1	Specific O.R.	7.6634	0.0760	0.0719	23.37	-0.0041
2	*	2	2-RGK-067-2	Specific O.R.	7.6433	0.0758	0.0717	23.43	-0.0041
3	*	3	2-RGK-067-3	Specific O.R.	7.5121	0.0745	0.0704	23.50	-0.0041
4	*	4	2-RGK-067-4	Specific O.R.	7.5626	0.0750	0.0709	23.56	-0.0041
5	*	5	2-RGK-067-5	Specific O.R.	7.5928	0.0753	0.0712	23.61	-0.0041
6	*	6	Avg.		7.5948				
7		7	S.D		0.0611				
8		8	C.V		0.8047				

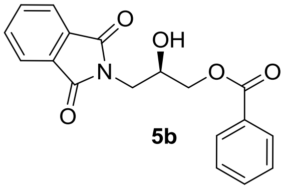

5b

SAMPLE INFORMATION

Sample Name:	R-related aldehy de	Acquired By:	Sy stem
Sample Ty pe:	Unknown	Date Acquired:	8/12/2010 6:40:20 PM
Vial:	88	Acq. Method Set:	CIRA2
Injection #:	1	Date Processed:	8/13/2010 11:28:23 AM
Injection Volume:	5.00 ul	Processing Methoc	cira2
Run Time:	40.0 Minutes	Channel Name:	Wv In Ch2
Sample Set Name	10081201	Proc. Chnl. Descr.	PDA 220.0 nm

Coloumn: ChiralPAK-IA(250x4.6mm,5.0u)
Mobile Phase: n-Hexane:ipa::80:20
Flow:1.0ml/min,Wav e Length:220nm,
Column Temperature:Ambient,Inj Vol:5ul,
Runtime:25min, Dilunt: mp, Conc:0.5mg/mL

Auto-Scaled Chromatogram

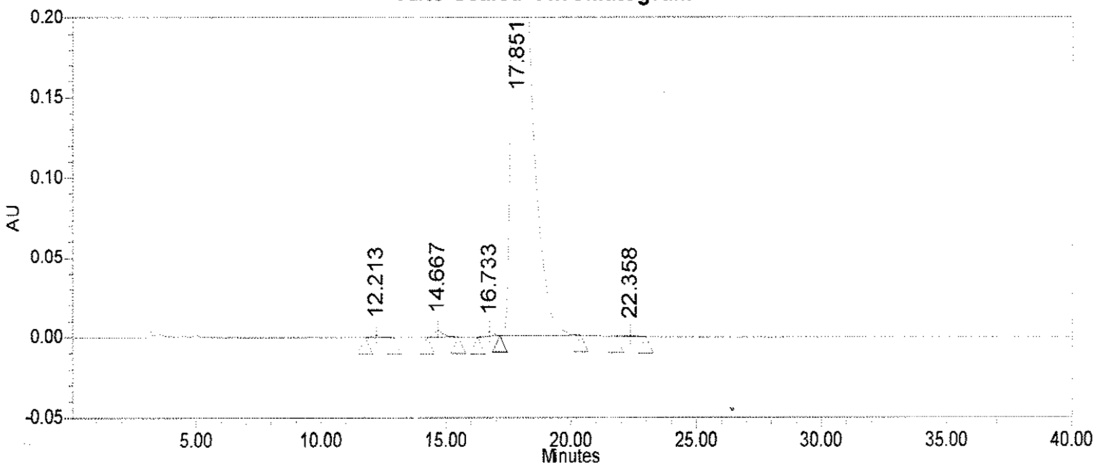

Peak Results

	Name	RT	Area	% Area
1		12.213	35169	0.13
2		14.667	102253	0.38
3		16.733	60435	0.22
4		17.851	26881191	99.21
5		22.358	15693	0.06

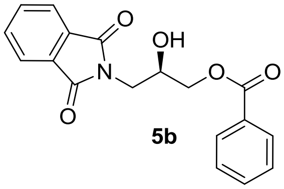

O
OH
N
O
O
O

5b

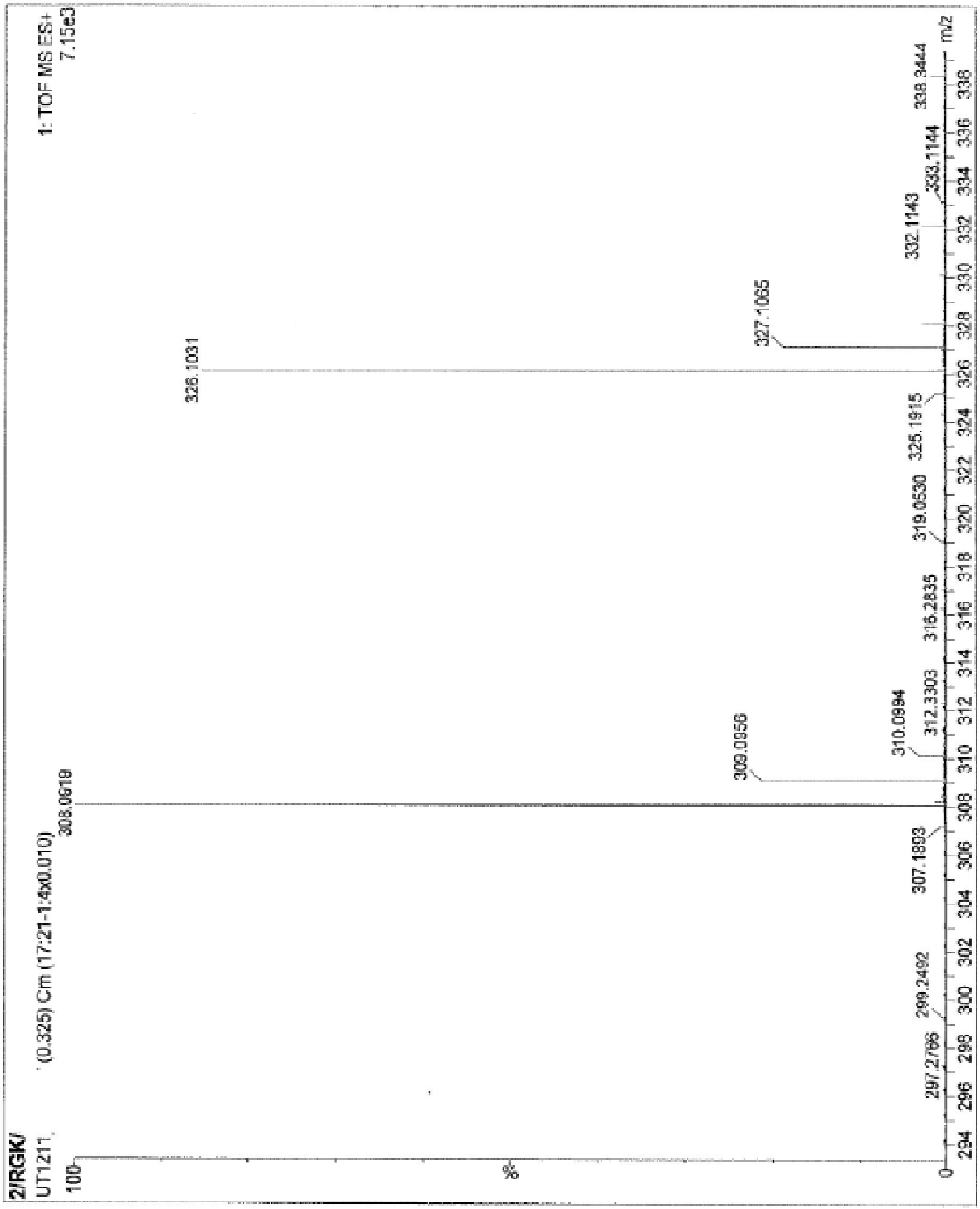

2/RGK/
UT1211
(0.325) Cm (17:21-1:4x0.010)
1: TOF MS ES+
7.15e3
100
%
0
308.0919
326.1031
327.1065
309.0956
310.0994
312.3303
316.2835
319.0530
325.1915
332.1143
333.1144
338.3444
307.1803
299.2492
297.2766
m/z

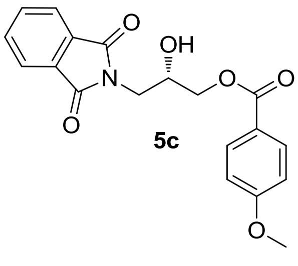

5c

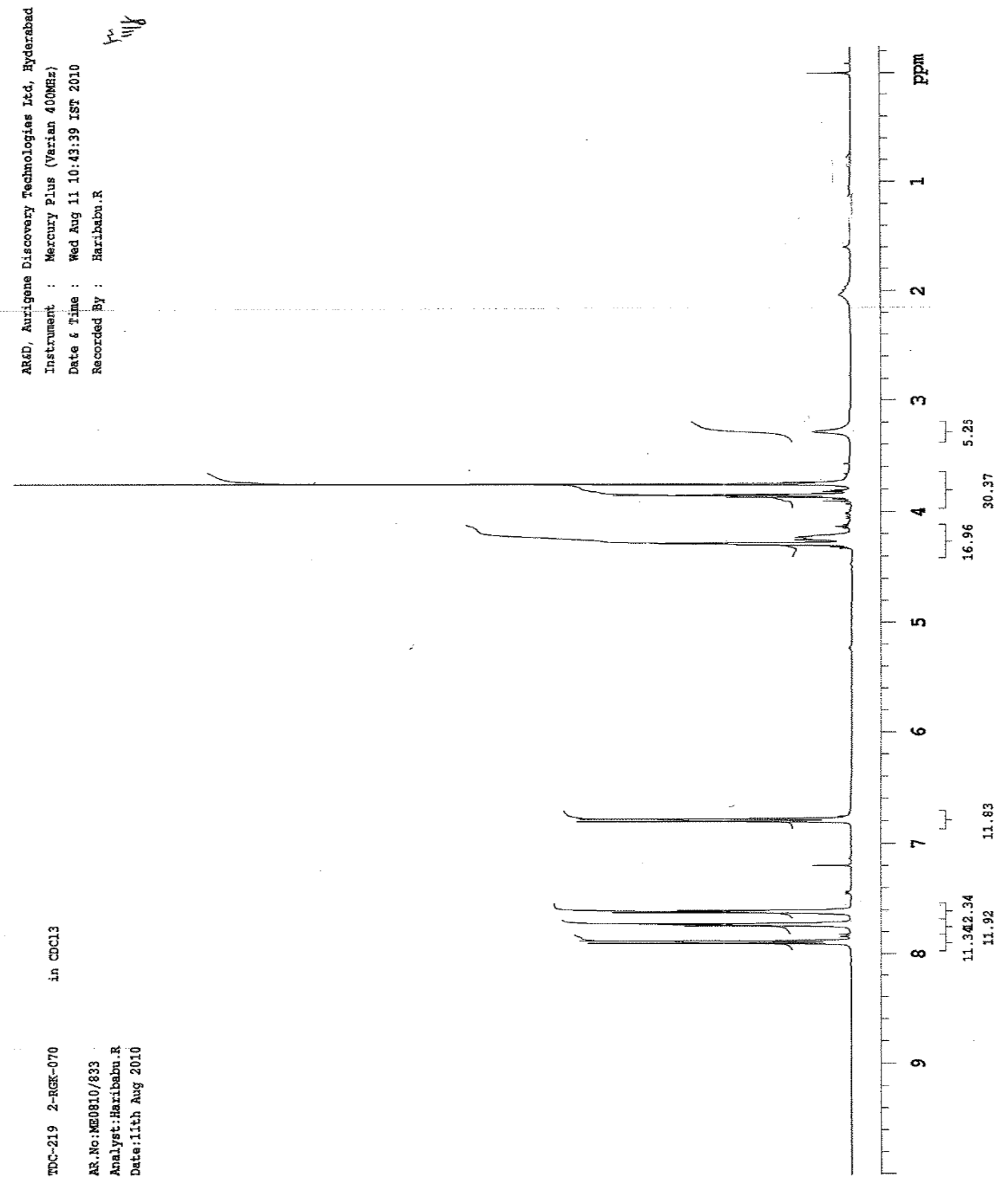

AR&D, Aurigene Discovery Technologies Ltd, Hyderabad
Instrument : Mercury Plus (Varian 400MHz)
Date & Time : Wed Aug 11 10:43:39 IST 2010
Recorded By : Haribabu.R
TDC-219 2-RGK-070 in CDCl3
AR.No:ME0810/833
Analyst:Haribabu.R
Date:11th Aug 2010
9 8 7 6 5 4 3 2 1 ppm
11.3412.34
11.92
11.83
16.96
30.37
5.25

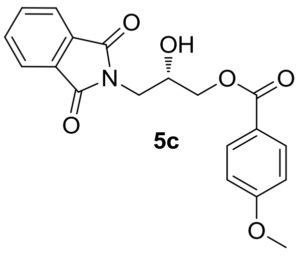

5c

AR&D, Aurigene Discovery Technologies Ltd, Hyderabad
Instrument : Mercury Plus (Varian 400MHz)
Date & Time : Fri Aug 13 15:18:52 IST 2010
Recorded By : Haribabu.R

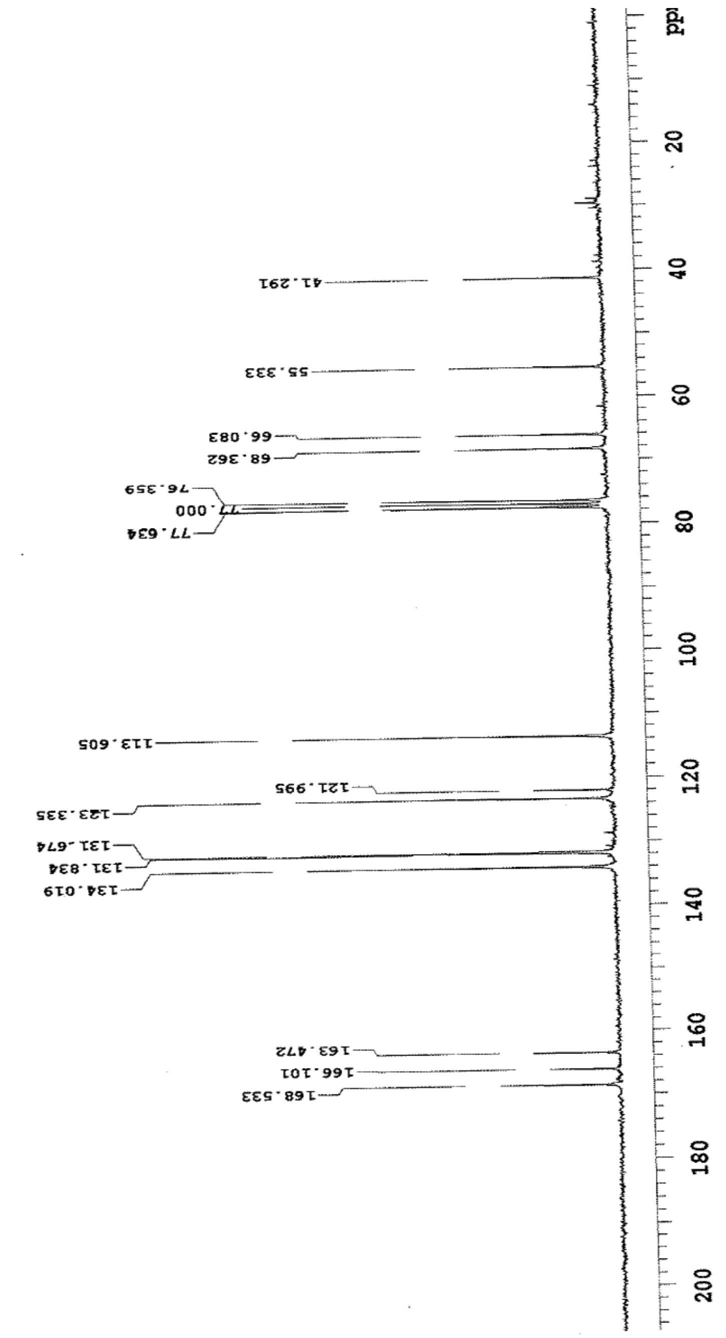

2-RGK-070 in CDCl3
TDC-219

AR NO:GE0810/24
Analyst:Haribabu.R
Date: 12 th Aug.2010

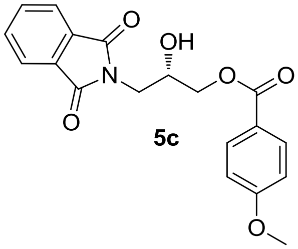

5c

2/RGK/072-S PURE-4OME

```
Injection Date  : 9/16/2010 5:40:25 PM          Seq. Line :   6
Sample Name     : 2/RGK/073                      Location : Vial 6
Acq. Operator   : Ashok                               Inj :   1
Acq. Instrument : CPS1/ARD/HPLC/003            Inj Volume : 5 µl
Acq. Method     : C:\HPCHEM\1\METHODS\CIRA2CH.M
Last changed    : 9/16/2010 5:32:47 PM by Ashok
                  (modified after loading)
Analysis Method : C:\HPCHEM\1\METHODS\CIRA2CH.M
Last changed    : 9/17/2010 8:33:52 AM by RAMANJANEYULU
                  (modified after loading)
Column: CHIRAL PAK IA(250*4.6)5µm
Mobile Phase :n-Hexane:IPA:8:2
Flow: 1.0mL/min, Wavelength: 220nm,
Run time:25min
```

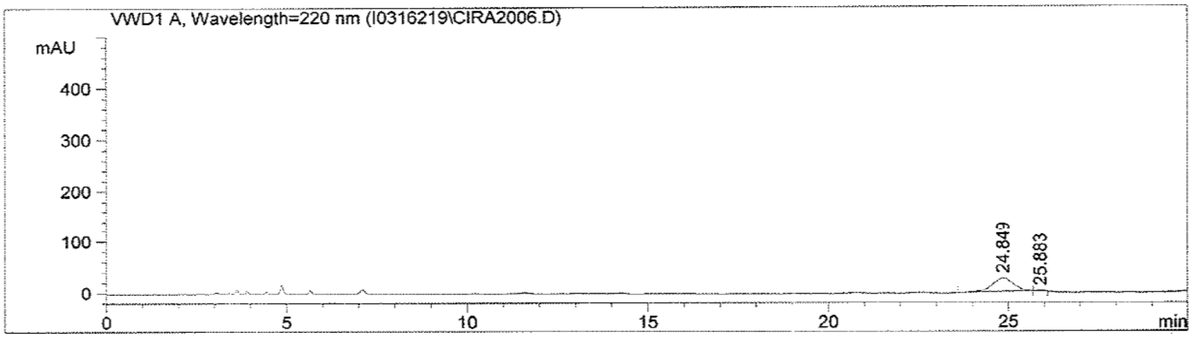

Area Percent Report

Sorted By	:	Signal
Multiplier	:	1.0000
Dilution	:	1.0000

Use Multiplier & Dilution Factor with ISTDs

Signal 1: VWD1 A, Wavelength=220 nm

Peak #	RetTime [min]	Type	Width [min]	Area mAU *s	Height [mAU]	Area %
1	24.849	MM R	0.9344	1084.80518	25.97355	98.3181
2	25.883	MM R	0.2241	18.55733	1.37995	1.6819
Totals :				1103.36251	27.35351	

Results obtained with enhanced integrator!

*** End of Report ***

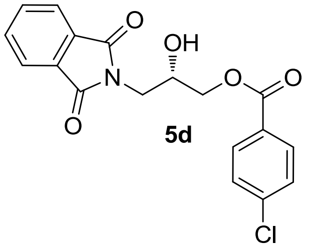
OH
O
N
O
O
O
Cl

5d

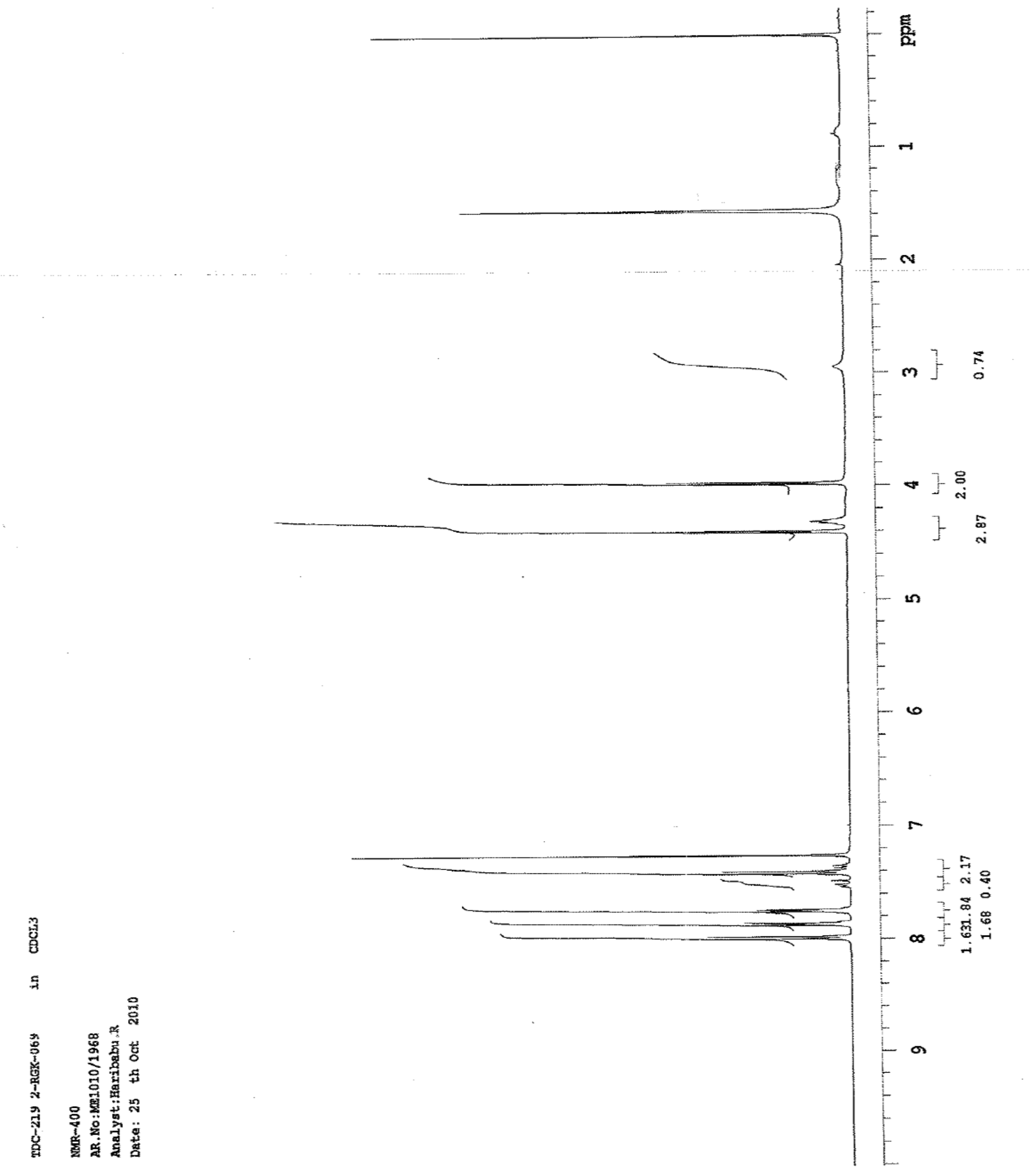
TDC-219 2-RGK-069 in CDCL3
NMR-400
AR.No:ME1010/1968
Analyst:Haribabu.R
Date: 25 th Oct 2010
ppm
0.74
2.00
2.87
1.631.84 2.17
1.68 0.40

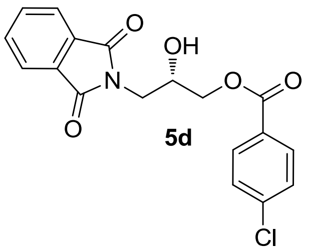
O
OH
N
O
O
O
Cl
5d

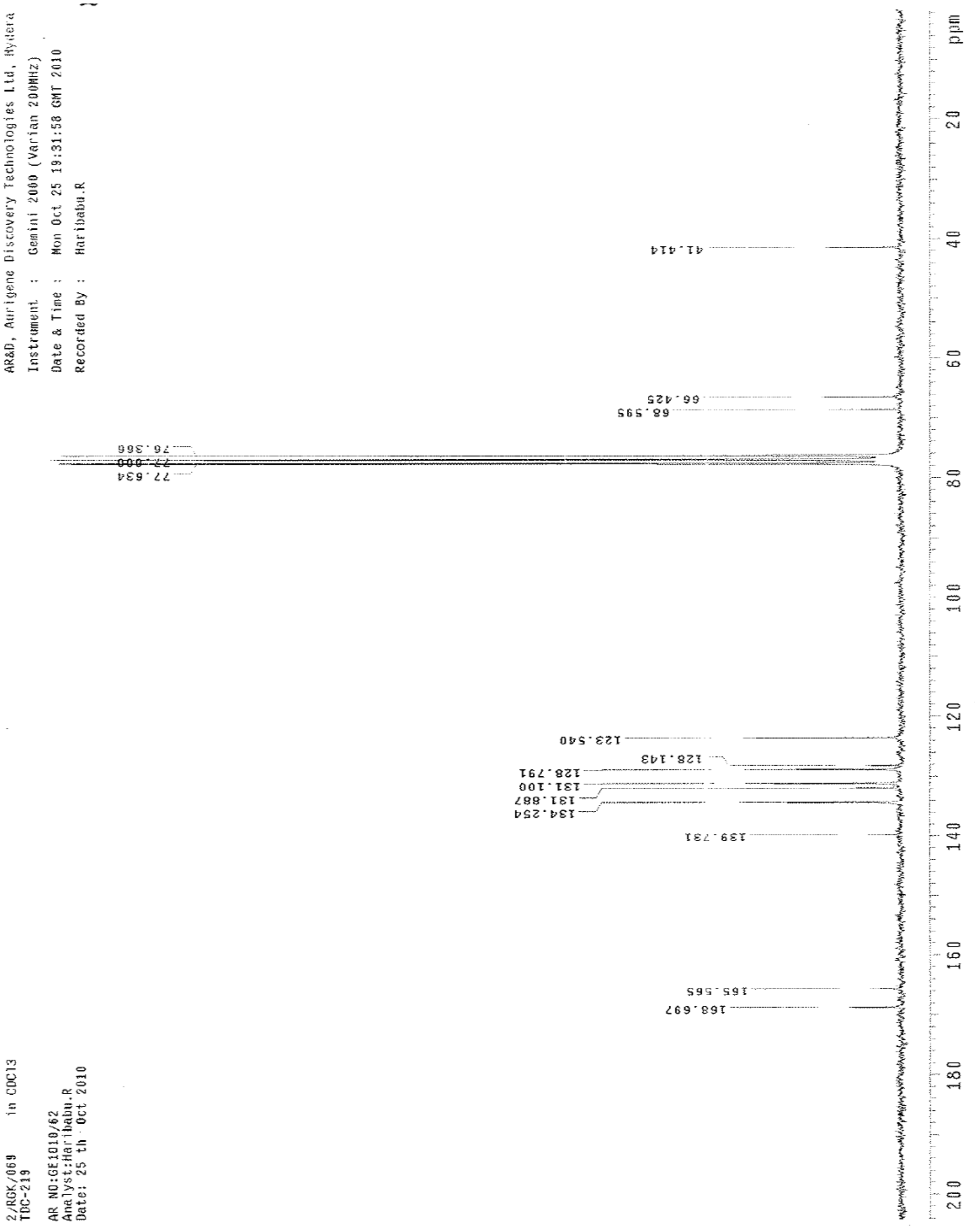
AR&D, Aurigene Discovery Technologies Ltd, Hydera
Instrument : Gemini 2000 (Varian 200MHz)
Date & Time : Mon Oct 25 19:31:58 GMT 2010
Recorded By : Haribabu.R
2/RGK/069 in CDCl3
TDC-219
AR NO:GE1010/62
Analyst:Haribabu.R
Date: 25 th Oct 2010
41.414
66.425
68.595
76.366
77.000
77.634
123.540
128.143
128.791
131.100
131.887
134.254
139.731
165.565
168.697
ppm

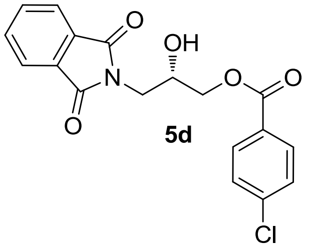

5d

```
=====================================================================
Injection Date  : 9/16/2010 4:42:07 PM           Seq. Line :   4
Sample Name     : 2/RGK/069                       Location : Vial 4
Acq. Operator   : Ashok                                Inj :   1
Acq. Instrument : CPS1/ARD/HPLC/003             Inj Volume : 5 µl
Acq. Method     : C:\HPCHEM\1\METHODS\CIRA2CH.M
Last changed    : 9/14/2010 8:10:18 PM by Muralikrishna Ch
Analysis Method : C:\HPCHEM\1\METHODS\CIRA2CH.M
Last changed    : 9/17/2010 8:33:52 AM by RAMANJANEYULU
                  (modified after loading)
Column: CHIRAL PAK IA(250*4.6)5µm
Mobile Phase :n-Hexane:IPA:8:2
Flow: 1.0mL/min, Wavelength: 220nm,
Run time:25min
```

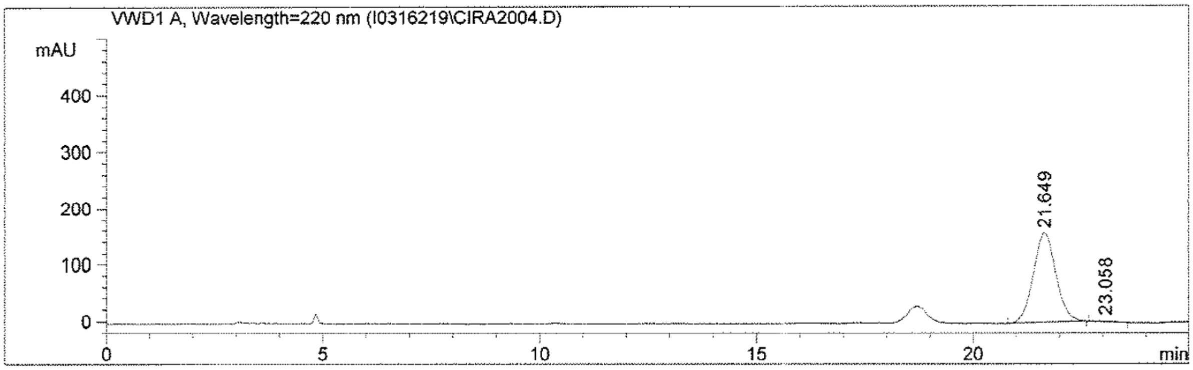


```
=====================================================================
                     Area Percent Report
=====================================================================

Sorted By             :      Signal
Multiplier            :      1.0000
Dilution              :      1.0000
Use Multiplier & Dilution Factor with ISTDs


Signal 1: VWD1 A, Wavelength=220 nm

Peak RetTime Type  Width     Area      Height     Area
  #   [min]        [min]  mAU    *s   [mAU   ]      %
----|-------|----|-------|----------|----------|--------|
   1  21.649 BB    0.5515 5608.70752  156.16954  99.5308
   2  23.058 MM R  0.4677   26.44208 9.42218e-1   0.4692

Totals :                  5635.14960  157.11176

 Results obtained with enhanced integrator!
=====================================================================
                     *** End of Report ***
```

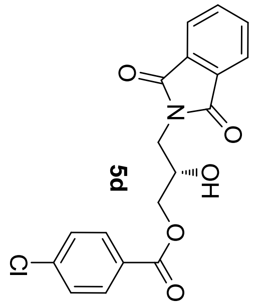

5d

Elemental Composition Report

Page 1

Single Mass Analysis
Tolerance = 5.0 PPM / DBE: min = -1.5, max = 80.0
Element prediction: Off
Number of isotope peaks used for i-FIT = 3

Monoisotopic Mass, Even Electron Ions
38 formula(e) evaluated with 1 results within limits (up to 4 best isotopic matches for each mass)
Elements Used:
C: 0-20 H: 0-20 N: 0-2 O: 0-6 Cl: 0-1
[illegible]
[illegible]

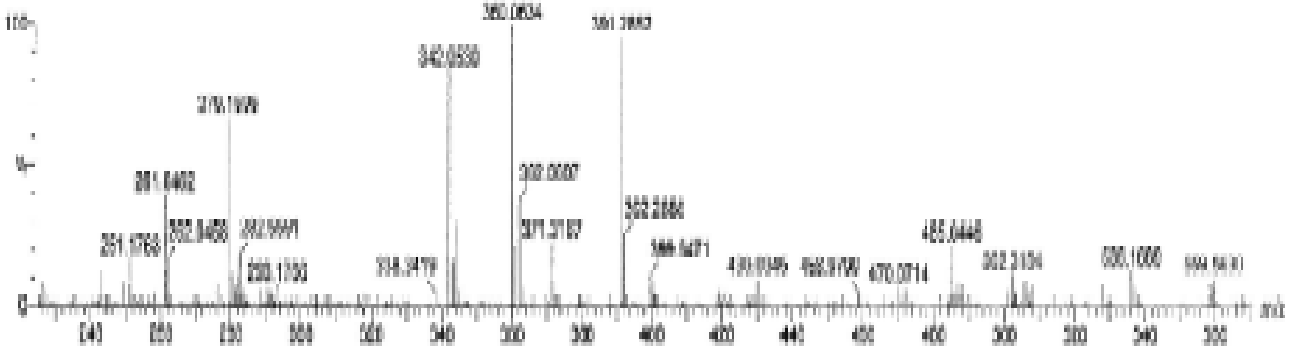

Minimum:				-1.5		
Maximum:		5.0	5.0	80.0		
Mass	Calc. Mass	mDa	PPM	DBE	i-FIT	Formula
360.0634	360.0639	-0.5	-1.4	11.5	2.2	C18 H15 N O5 Cl

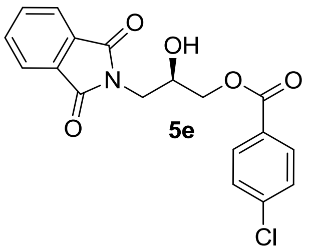

O
OH
N
O
O
O
Cl

5e

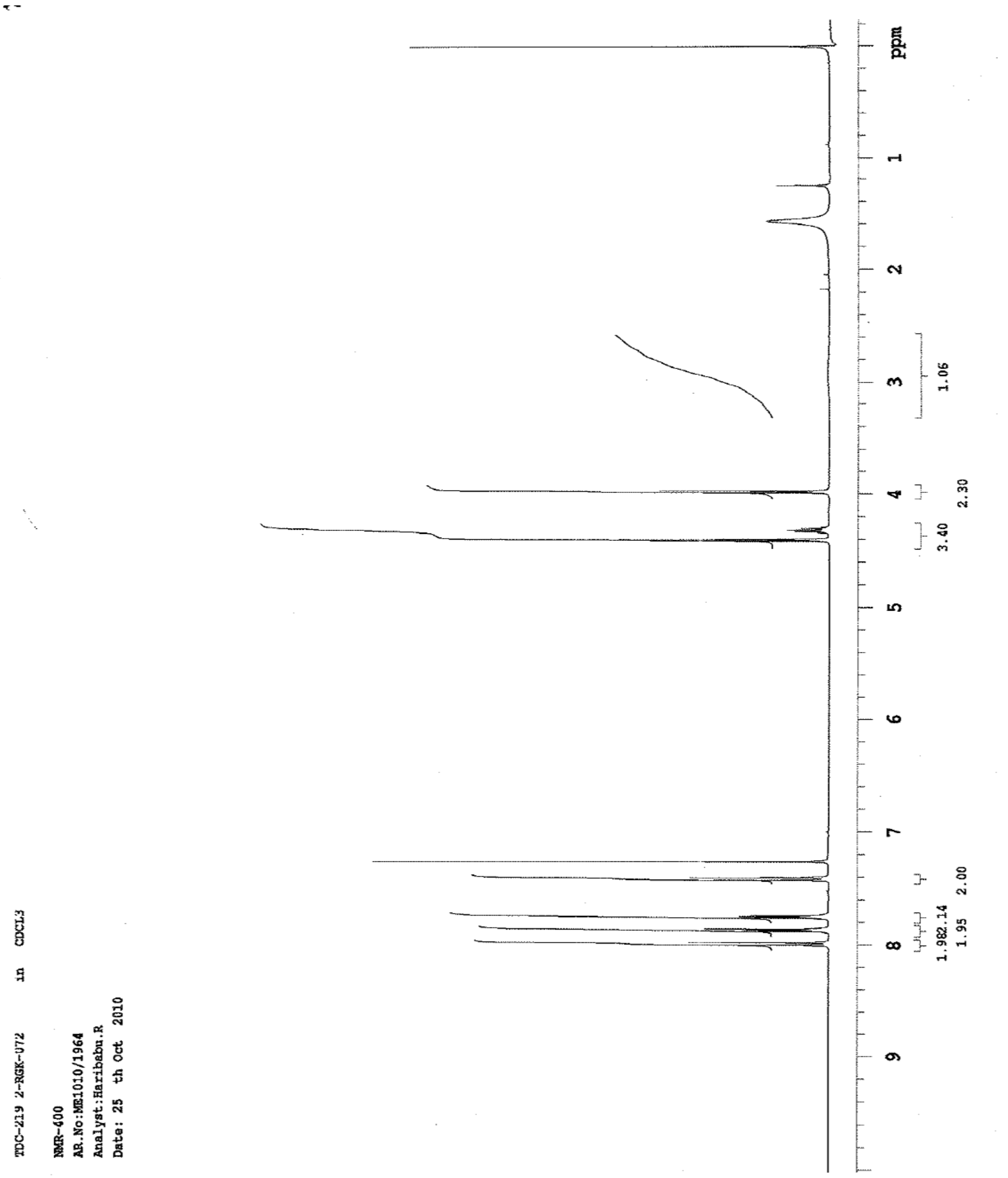

TDC-219 2-RGK-U72 in CDCL3
NMR-400
AR.No:ME1010/1964
Analyst:Haribabu.R
Date: 25 th Oct 2010
ppm
1
2
3
4
5
6
7
8
9
1.06
2.30
3.40
2.00
1.982.14
1.95

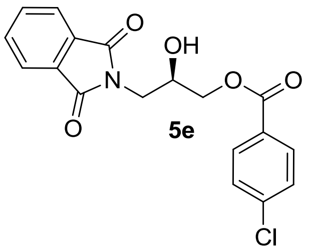
O
OH
N
O
O
O
5e
Cl

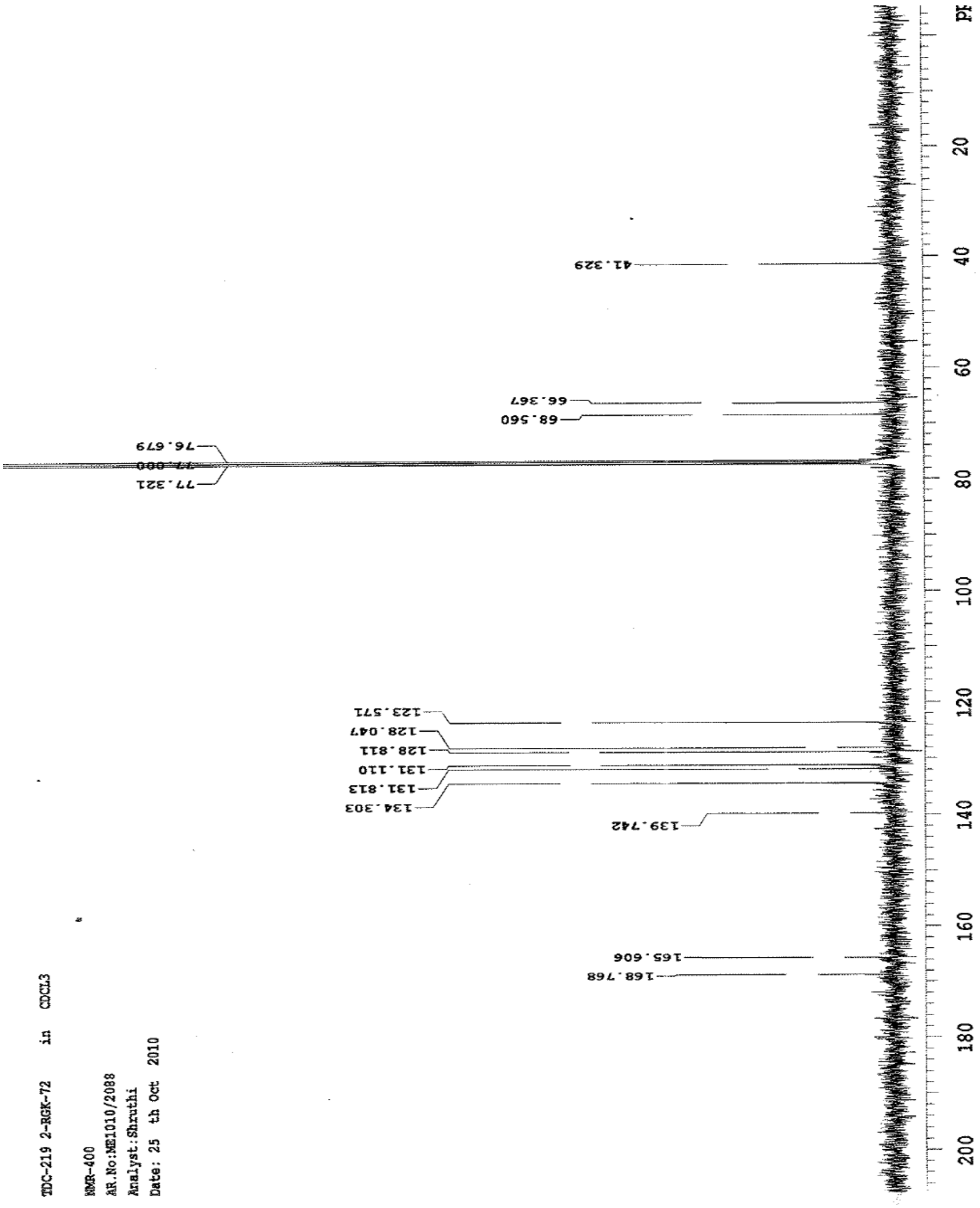
TDC-219 2-RGK-72 in CDCL3
NMR-400
AR.No:ME1010/2088
Analyst:Shruthi
Date: 25 th Oct 2010
41.329
66.367
68.560
76.679
77.000
77.321
123.571
128.047
128.811
131.110
131.813
134.303
139.742
165.606
168.768
20
40
60
80
100
120
140
160
180
200

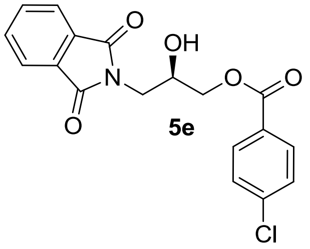

5e

```
=====================================================================
Injection Date  : 9/16/2010 5:08:47 PM            Seq. Line :    5
Sample Name     : 2/RGK/072                        Location : Vial 5
Acq. Operator   : Ashok                                 Inj :    1
Acq. Instrument : CPS1/ARD/HPLC/003              Inj Volume : 5 µl
Acq. Method     : C:\HPCHEM\1\METHODS\CIRA2CH.M
Last changed    : 9/16/2010 5:32:47 PM by Ashok
                  (modified after loading)
Analysis Method : C:\HPCHEM\1\METHODS\CIRA2CH.M
Last changed    : 9/17/2010 8:33:52 AM by RAMANJANEYULU
                  (modified after loading)
Column: CHIRAL PAK IA(250*4.6)5µm
Mobile Phase :n-Hexane:IPA:8:2
Flow: 1.0mL/min, Wavelength: 220nm,
Run time:25min
```

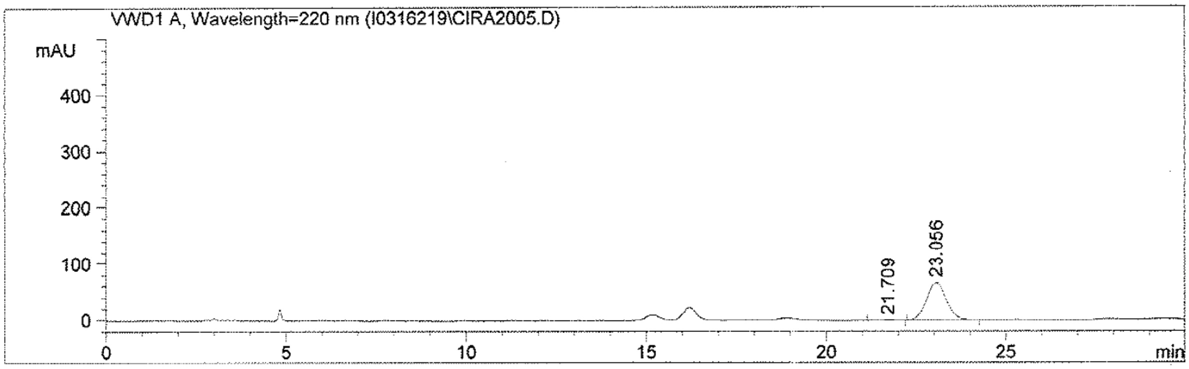


```
=====================================================================
                         Area Percent Report
=====================================================================

Sorted By             :      Signal
Multiplier            :      1.0000
Dilution              :      1.0000
Use Multiplier & Dilution Factor with ISTDs


Signal 1: VWD1 A, Wavelength=220 nm

Peak RetTime Type  Width     Area      Height     Area
  #   [min]        [min]  mAU   *s    [mAU   ]     %
----|-------|----|-------|----------|----------|--------|
   1  21.709 MM R  0.5429   21.54136 6.61289e-1  0.8460
   2  23.056 MM R  0.6389 2524.79077   65.86342  99.1540

Totals :                  2546.33213   66.52471

 Results obtained with enhanced integrator!
=====================================================================
                         *** End of Report ***
```

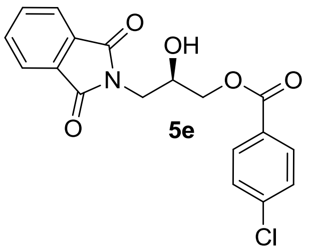
O
OH
N
O
O
O
Cl

5e

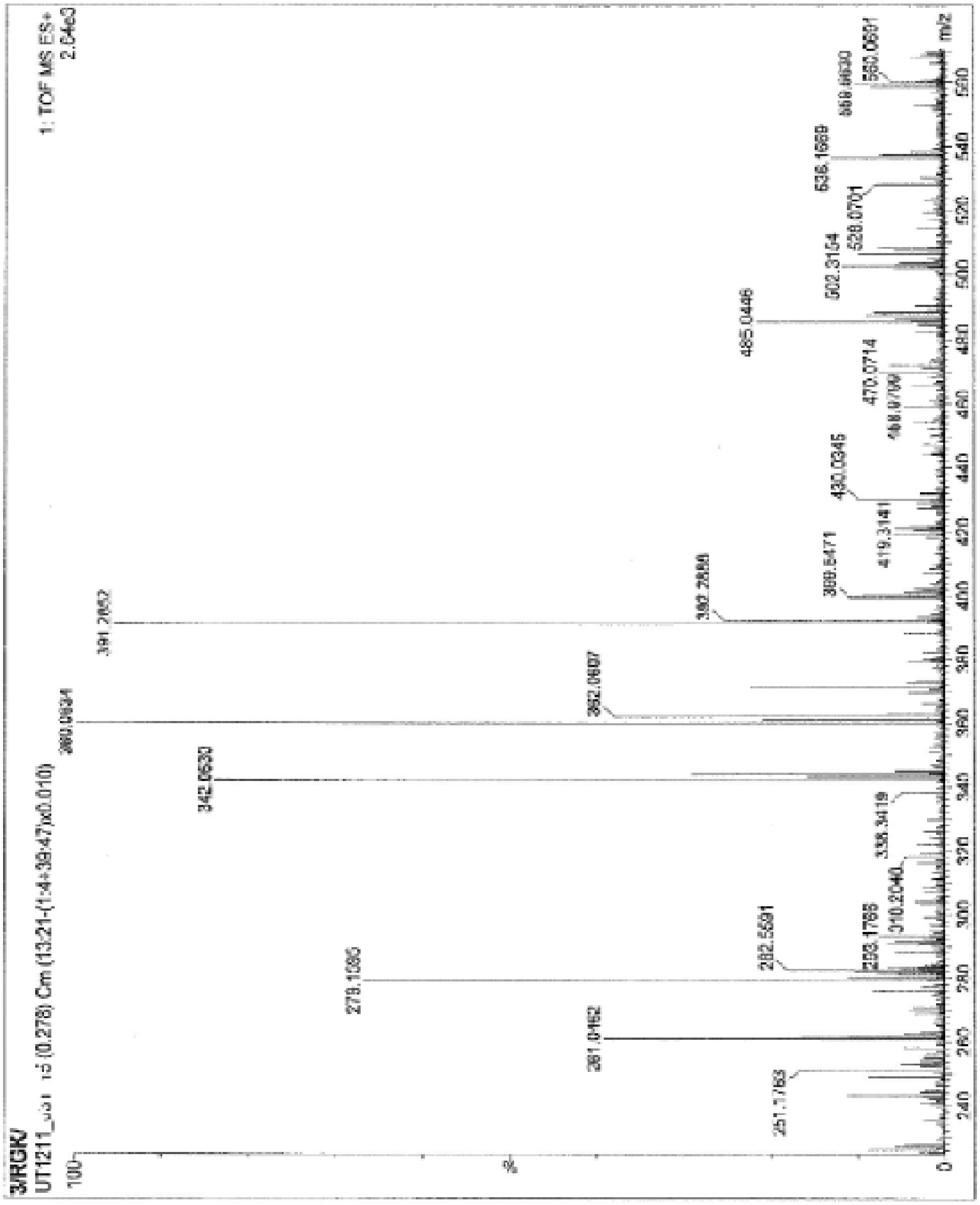
1: TOF MS ES+
2.04e3
100
%
0
m/z
251.1763
261.0162
279.1080
282.5591
288.1765
310.2040
338.3419
342.0630
360.0694
362.0607
391.2852
392.2888
399.6471
419.3141
430.0345
470.0714
485.0446
502.3154
528.0701
536.1669
559.0691
240
260
280
300
320
340
360
380
400
420
440
460
480
500
520
540
560

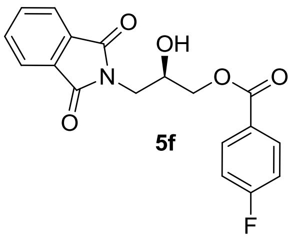

5f

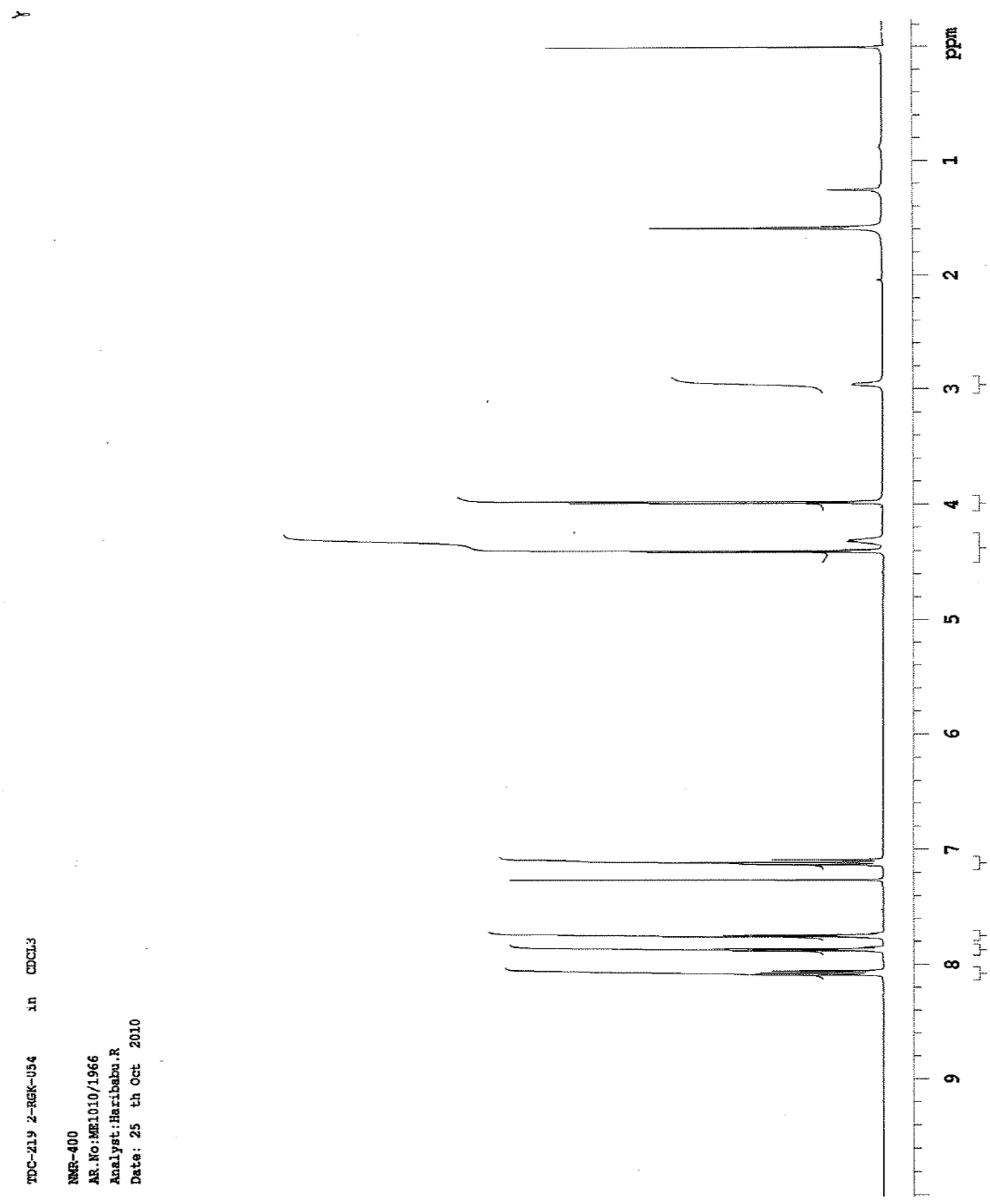
TDC-219 2-RGK-U54 in CDCL3
NMR-400
AR.No:ME1010/1966
Analyst:Haribabu.R
Date: 25 th Oct 2010
ppm
0.94
3.33
2.00
1.96 2.07

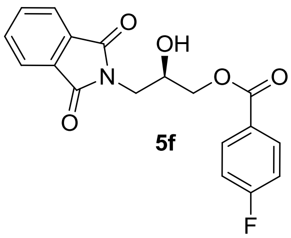
O
OH
N
O
O
O
F

5f

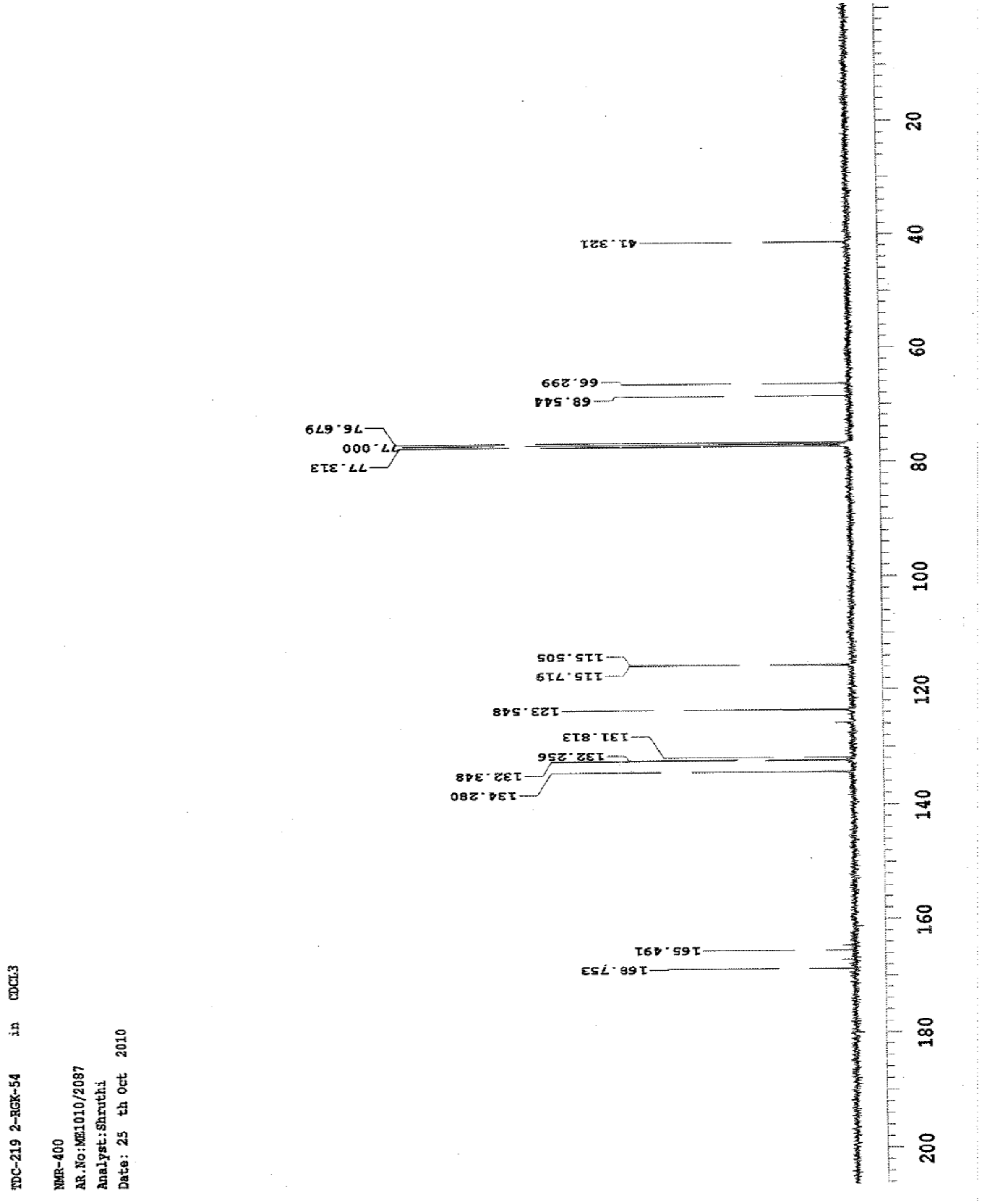
TDC-219 2-RGK-54 in CDCL3
NMR-400
AR.No:ME1010/2087
Analyst:Shruthi
Date: 25 th Oct 2010
41.321
66.299
68.544
76.679
77.000
77.313
115.505
115.719
123.548
131.813
132.256
132.348
134.280
165.491
168.753
20
40
60
80
100
120
140
160
180
200

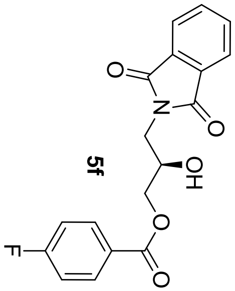

5f

Elemental Composition Report

Page 1

Single Mass Analysis
Tolerance = 5.0 PPM / DBE: min = -1.5, max = 80.0
Element prediction: Off
Number of isotope peaks used for i-FIT = 3

Monoisotopic Mass, Even Electron Ions
75 formula(e) evaluated with 1 results within limits (up to 4 best isotopic matches for each mass)
Elements Used:
C: 0-20 H: 0-20 N: 0-4 O: 0-6 F: 0-1

3/RGK/064
U11211_029 15 (0.279) Cm (15:20)

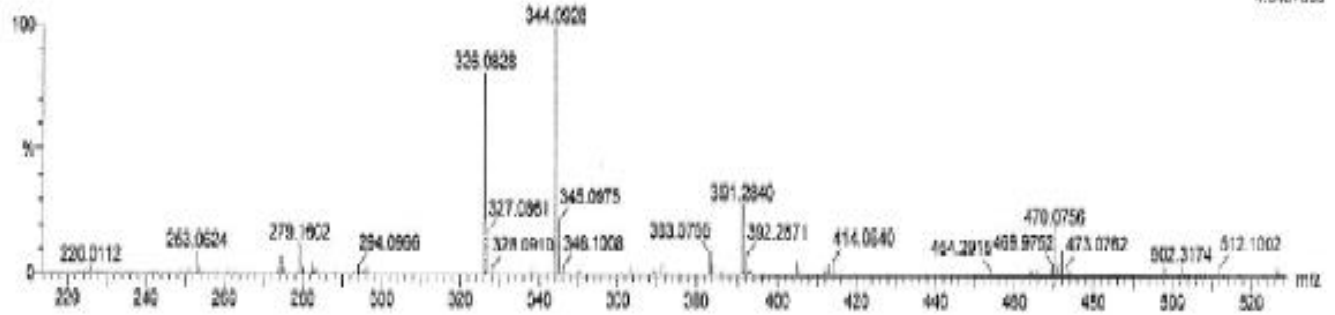

Minimum:				-1.5		
Maximum:		5.0	5.0	80.0		
Mass	Calc. Mass	mDa	PPM	DBE	i-FIT	Formula
344.0928	344.0934	-0.6	-1.7	11.5	3.0	C10 H15 N O6 7

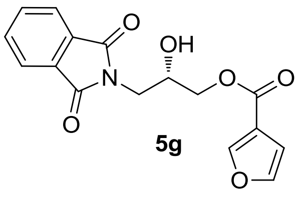

5g

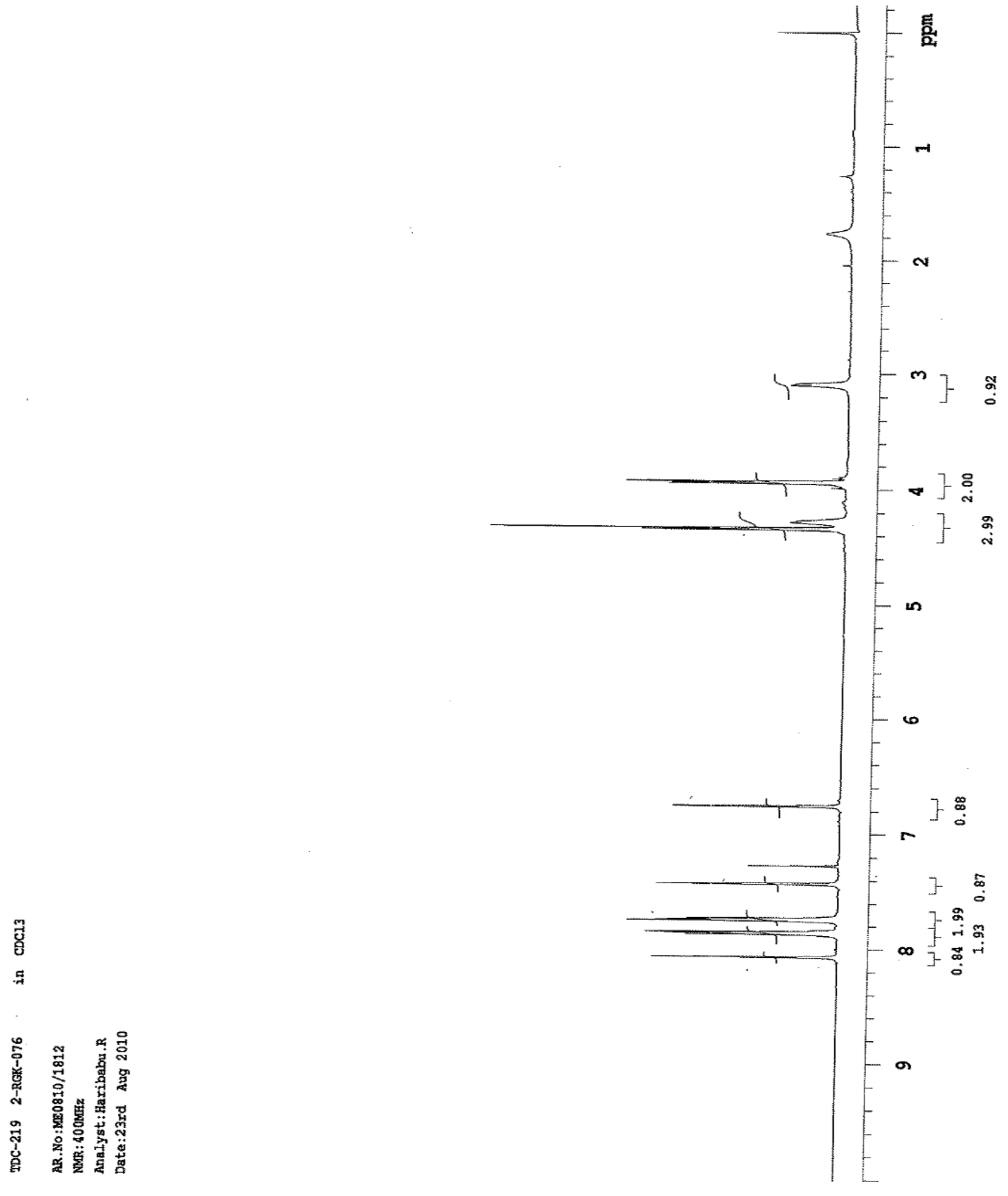

TDC-219 2-RGK-076 in CDCl3
AR.No:ME0810/1812
NMR:400MHz
Analyst:Haribabu.R
Date:23rd Aug 2010
ppm
0.92
2.00
2.99
0.88
0.87
0.84 1.99
1.93

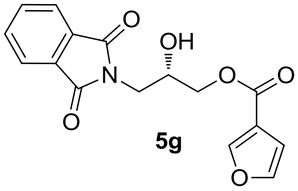

5g

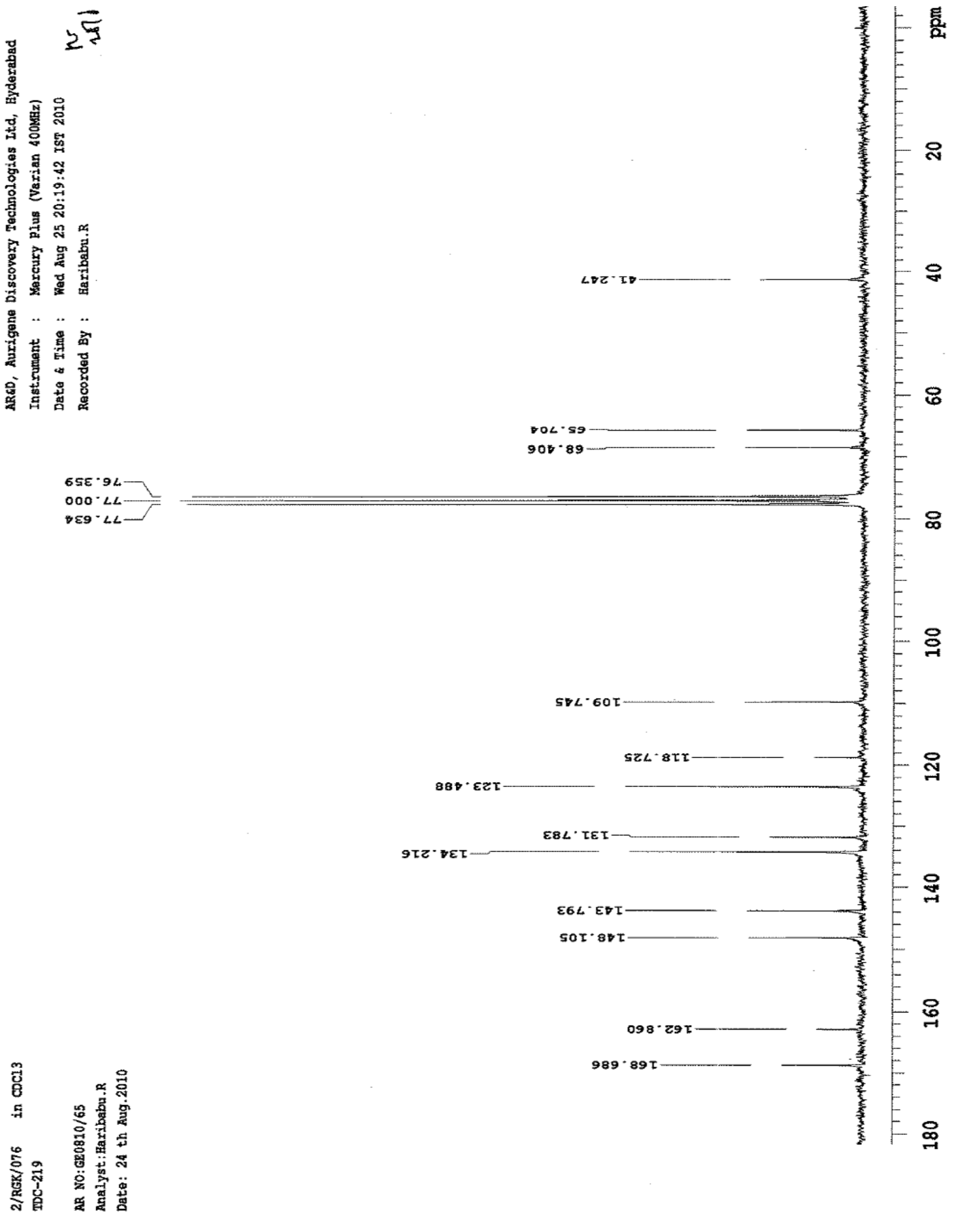
AR&D, Aurigene Discovery Technologies Ltd, Hyderabad
Instrument : Mercury Plus (Varian 400MHz)
Date & Time : Wed Aug 25 20:19:42 IST 2010
Recorded By : Haribabu.R
2/RGK/076 in CDCl3
TDC-219
AR NO:GE0810/65
Analyst:Haribabu.R
Date: 24 th Aug.2010
41.247
65.704
68.406
76.359
77.000
77.634
109.745
118.725
123.488
131.783
134.216
143.793
148.105
162.860
168.686
20
40
60
80
100
120
140
160
180
ppm

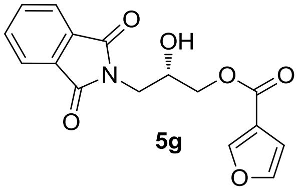

5g

```
=====================================================================
Injection Date  : 9/16/2010 3:48:43 PM           Seq. Line :   2
Sample Name     : 2/RGK/076 FURAN                 Location : Vial 2
Acq. Operator   : Ashok                                Inj :   1
Acq. Instrument : CPS1/ARD/HPLC/003            Inj Volume : 5 µl
Acq. Method     : C:\HPCHEM\1\METHODS\CIRA2CH.M
Last changed    : 9/14/2010 8:10:18 PM by Muralikrishna Ch
Analysis Method : C:\HPCHEM\1\METHODS\CIRA2CH.M
Last changed    : 9/17/2010 8:33:52 AM by RAMANJANEYULU
                  (modified after loading)
Column: CHIRAL PAK IA(250*4.6)5µm
Mobile Phase :n-Hexane:IPA:8:2
Flow: 1.0mL/min, Wavelength: 220nm,
Run time:25min
```

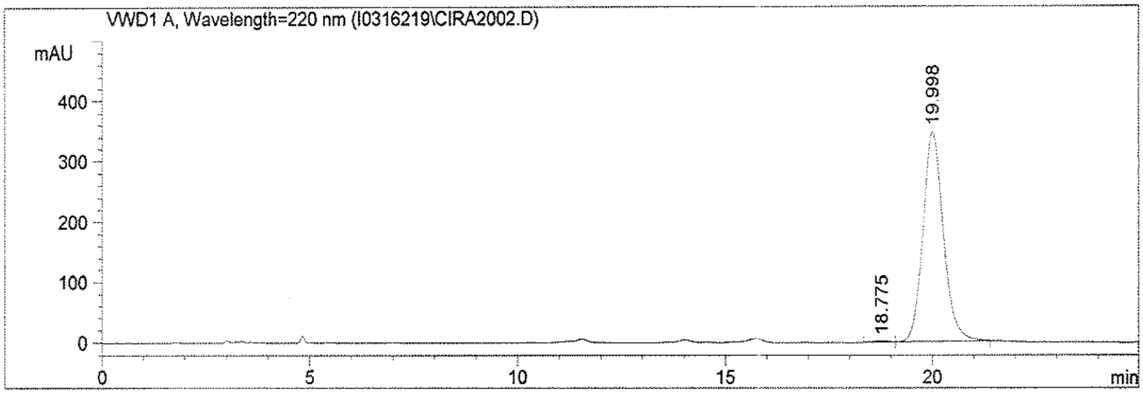


```
=====================================================================
                         Area Percent Report
=====================================================================

Sorted By             :      Signal
Multiplier            :      1.0000
Dilution              :      1.0000
Use Multiplier & Dilution Factor with ISTDs


Signal 1: VWD1 A, Wavelength=220 nm

Peak RetTime Type  Width     Area      Height     Area
  #   [min]        [min]  mAU   *s   [mAU   ]       %
----|-------|----|-------|----------|----------|--------|
   1  18.775 BV    0.3991   44.23079    1.66289   0.3706
   2  19.998 VB    0.5186 1.18905e4   347.51443  99.6294

Totals :                  1.19347e4   349.17732

 Results obtained with enhanced integrator!
=====================================================================
                         *** End of Report ***
```

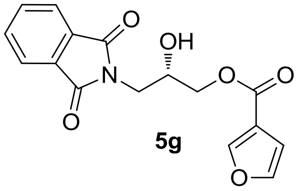

5g

2-RGK-076.p1d

[Comment]
Sample name
Comment
User ramanjaneyulu
Workgroup ARND
Division
Company DRL

[Data Information]
Creation Date 10/1/2010 12:25 PM

[Measurement Information]
Instrument Name PL/118/002
Model Name P-2000
Serial No. A035861232
Polarizer Glan-Taylor Prism
Faraday Cell Flint Glass

Accessory PTC-203
Accessory S/N A014261234
Path Length 100 mm

Light Source Na
Monitor wavelength 589 nm
D.I.T. 5 sec
No. of cycle 5
Cycle interval 0 sec
Temp. Monitor Cell
Temp. Corr. Factor None
Aperture(S) 3.0mm
Aperture(L) Auto
Mode Specific O.R.
Path Length 100 mm
Concentration 0.6139 w/v
Factor 1

		No.	Sample No.	Mode	Calc. Data	Meas. Data	Monitor(deg)	Temperature(C)	Blank
1	*	1	2-RGK-076-1	Specific O.R.	-10.1515	-0.0623	-0.0538	24.66	0.0085
2	*	2	2-RGK-076-2	Specific O.R.	-9.4836	-0.0582	-0.0497	24.65	0.0085
3	*	3	2-RGK-076-3	Specific O.R.	-9.3370	-0.0573	-0.0488	24.64	0.0085
4	*	4	2-RGK-076-4	Specific O.R.	-9.3859	-0.0576	-0.0491	24.64	0.0085
5	*	5	2-RGK-076-5	Specific O.R.	-9.0927	-0.0558	-0.0473	24.63	0.0085
6	*	6	Avg.		-9.4901				
7		7	S.D		0.3968				
8		8	C.V		4.1809				

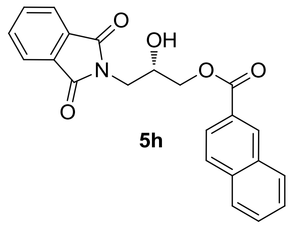

5h

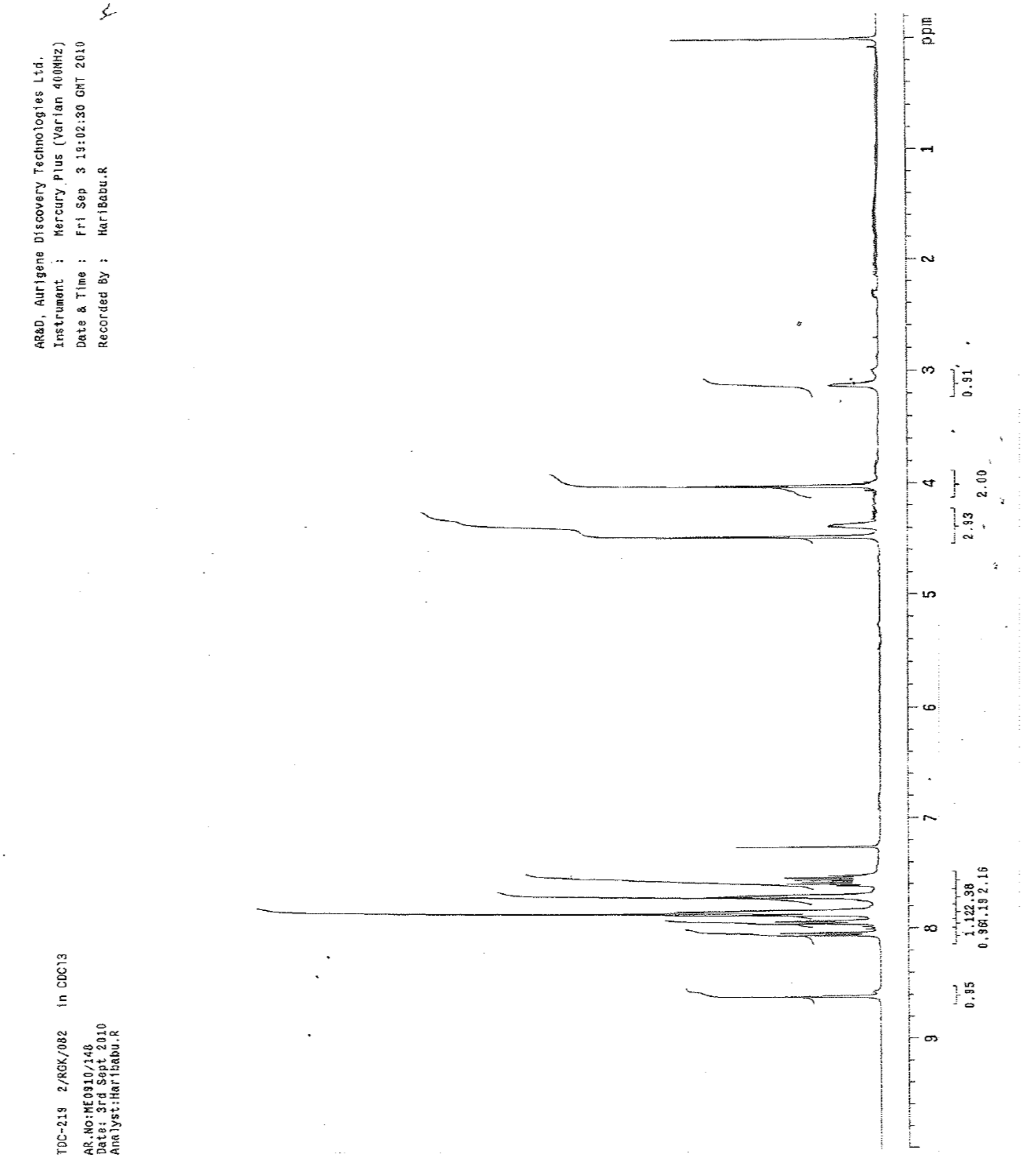
AR&D, Aurigene Discovery Technologies Ltd.
Instrument : Mercury Plus (Varian 400MHz)
Date & Time : Fri Sep 3 19:02:30 GMT 2010
Recorded By : HariBabu.R
TDC-219 2/RGK/082 in CDCl3
AR.NO:ME0910/148
Date: 3rd Sept 2010
Analyst:Haribabu.R
ppm
1
2
3
4
5
6
7
8
9
0.91
2.00
2.93
0.95

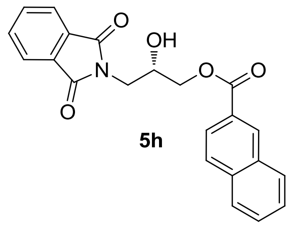
O
OH
N
O
O
O

5h

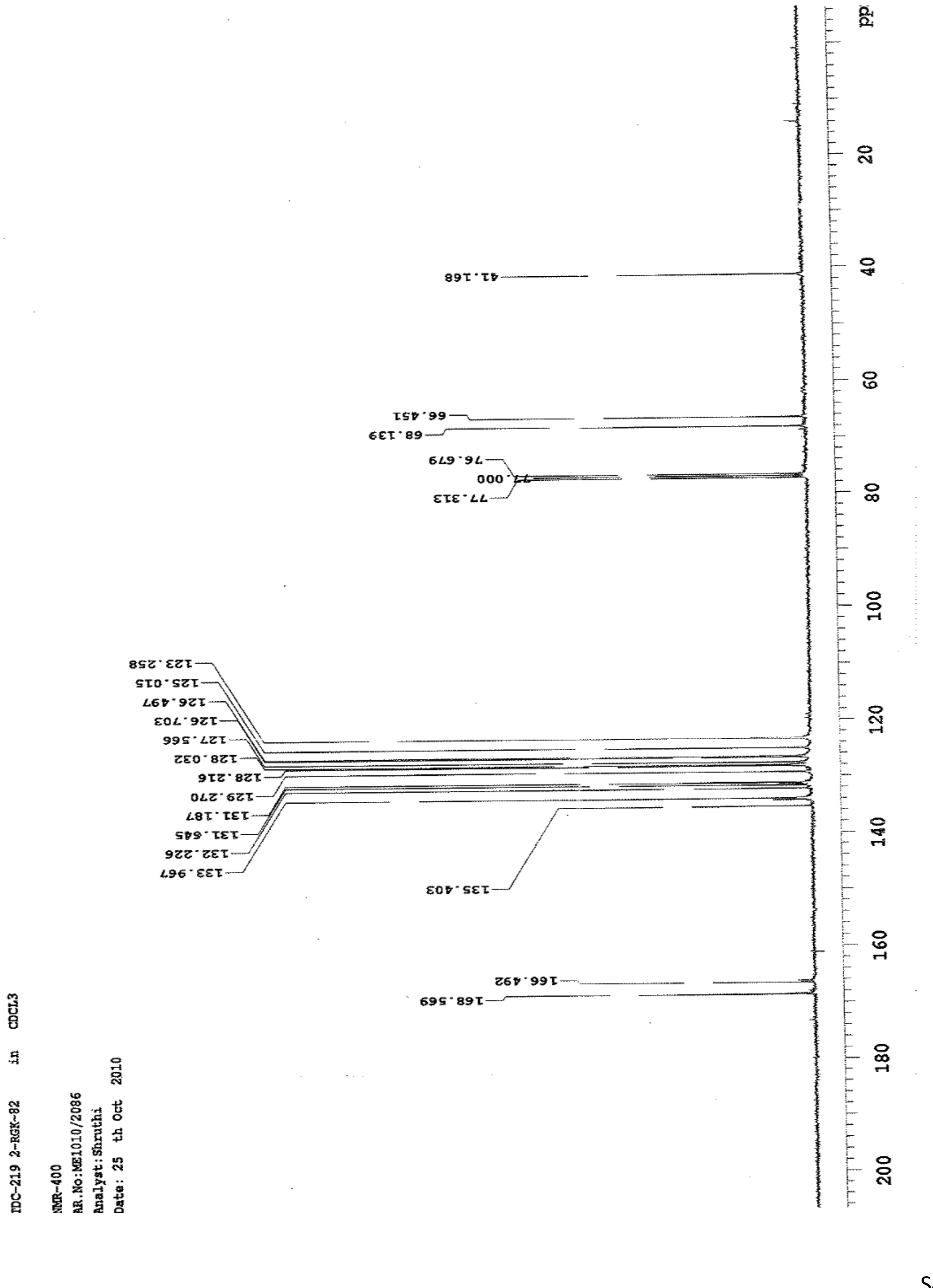
IDC-219 2-RGK-82 in CDCL3
NMR-400
AR.No:ME1010/2086
Analyst:Shruthi
Date: 25 th Oct 2010
41.168
66.451
68.139
76.679
77.000
77.313
123.258
125.015
126.497
126.703
127.566
128.032
128.216
129.270
131.187
131.645
132.226
133.967
135.403
166.492
168.569
20
40
60
80
100
120
140
160
180
200

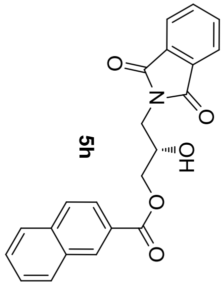

5h

Page 1

Elemental Composition Report

Single Mass Analysis

Tolerance = 5.0 PPM / DBE: min = -1.5, max = 80.0
Element prediction: Off
Number of isotope peaks used for i-FIT = 3

Monoisotopic Mass, Even Electron Ions
130 formula(e) evaluated with 1 results within limits (up to 4 best isotopic matches for each mass)
Elements Used:
C: 0-30 H: 0-30 N: 0-6 O: 0-6
3/rgk/082
UT1211_081 13 (0.281) Cm (13:28-1.4x0.010)

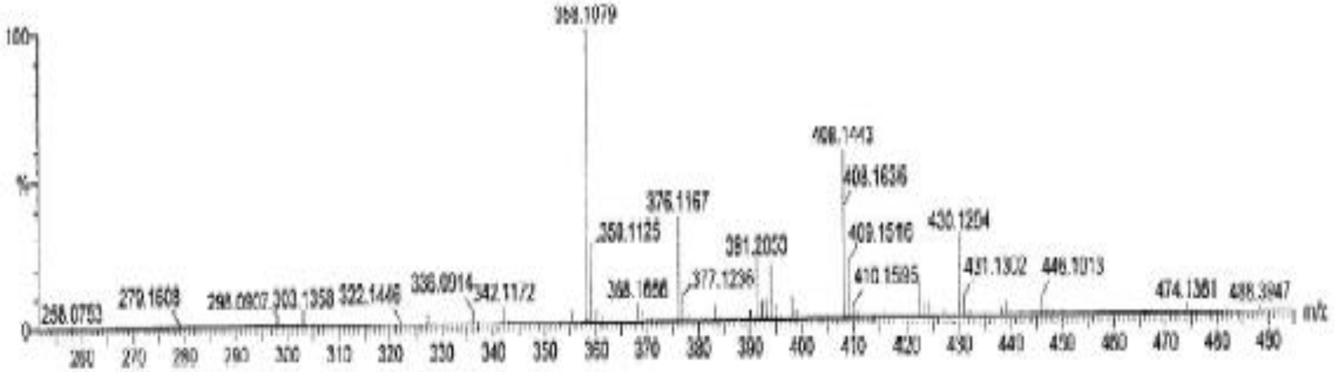

Minimum:				-1.5		
Maximum:		5.0	5.0	80.0		
Mass	Calc. Mass	mDa	PPM	DBE	i-FIT	Formula
376.1167	376.1185	-1.8	-4.0	14.5	3.0	C22 H19 N O5

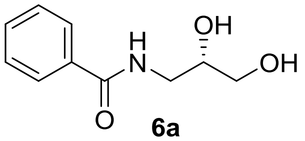

6a

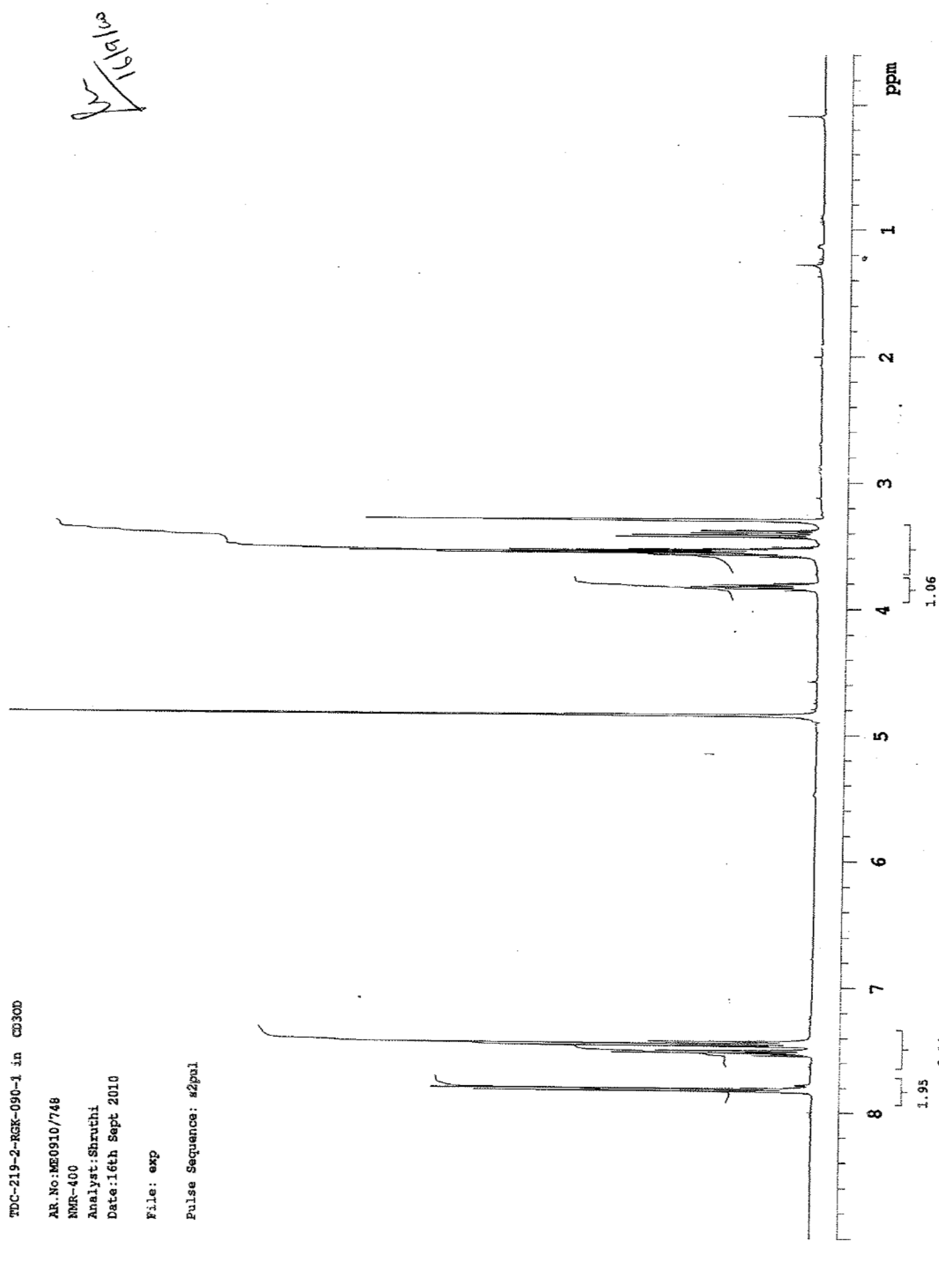
TDC-219-2-RGK-090-1 in CD3OD
AR.No:ME0910/748
NMR-400
Analyst:Shruthi
Date:16th Sept 2010
File: exp
Pulse Sequence: s2pul
ppm
1
2
3
4
5
6
7
8
1.06
4.53
3.14
1.95

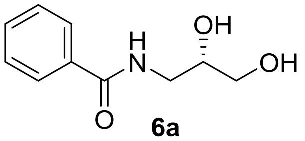
OH
H
N
OH
O

6a

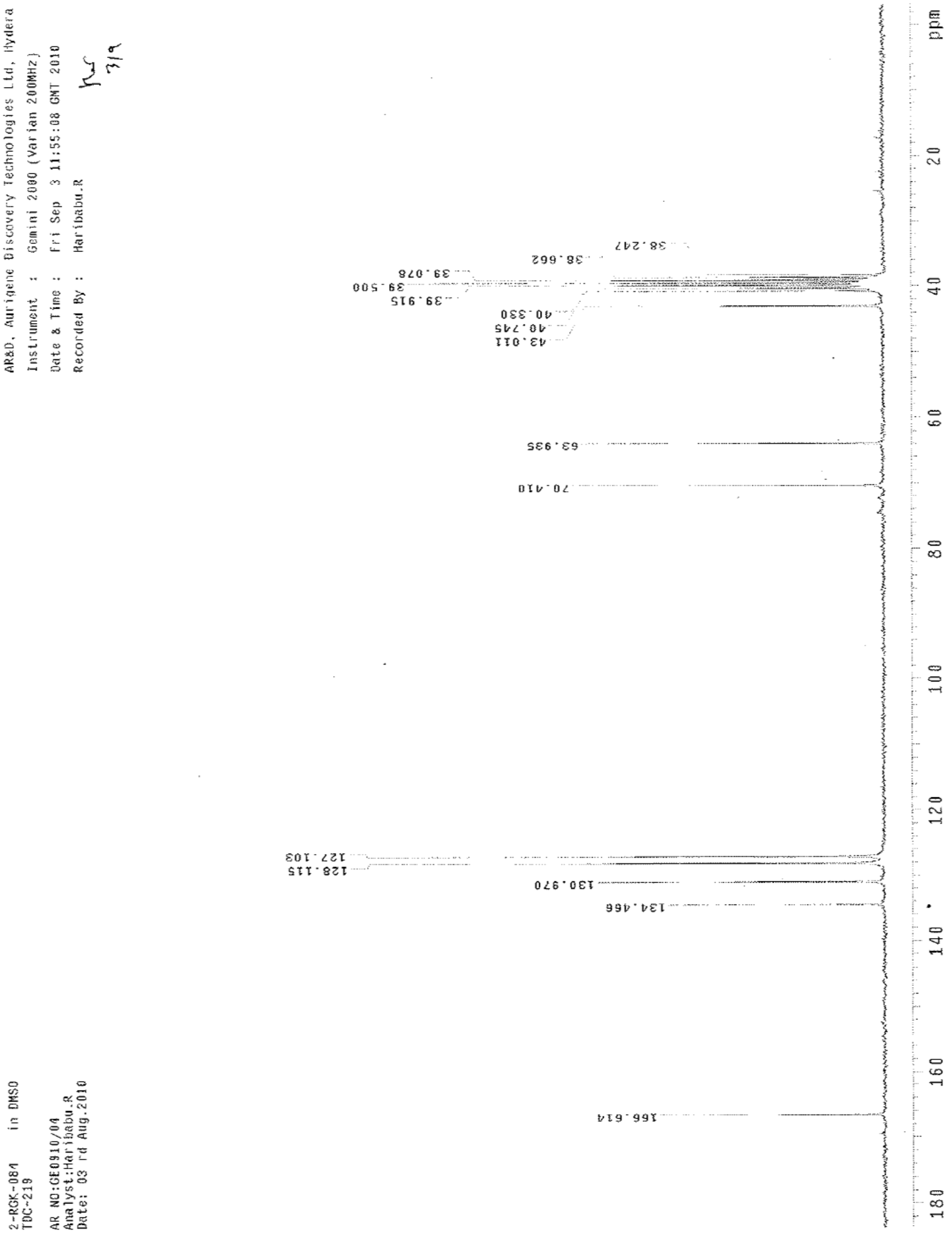
AR&D, Aurigene Discovery Technologies Ltd, Hydera
Instrument : Gemini 2000 (Varian 200MHz)
Date & Time : Fri Sep 3 11:55:08 GMT 2010
Recorded By : Haribabu.R
2-RGK-084 in DMSO
TDC-219
AR NO:GE0910/04
Analyst:Haribabu.R
Date: 03 rd Aug.2010
38.247
38.662
39.078
39.500
39.915
40.330
40.745
43.011
63.935
70.410
127.103
128.115
130.970
134.466
166.614
ppm
20
40
60
80
100
120
140
160
180

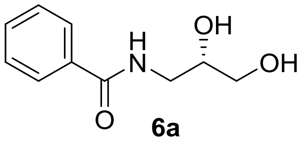

6a

SAMPLE INFORMATION

Sample Name:	2/RGK/090	Acquired By:	System
Sample Type:	Unknown	Date Acquired:	10/1/2010 1:21:38 PM
Vial:	27	Acq. Method Set:	CIRA2
Injection #:	1	Date Processed:	10/11/2010 5:11:16 PM
Injection Volume:	10.00 ul	Processing Methoc	cira2
Run Time:	40.0 Minutes	Channel Name:	Wvln Ch2
Sample Set Name	10100101	Proc. Chnl. Descr.	PDA 220.0 nm

Coloumn: Chiral pak IA(250x4.6mm,5µ)
Mobile Phase:N-HEXANE:IPA(8:2)
Flow:1.0ml/min,Wave Length:220nm,
Column Temperature:40°C,Inj Vol:10ul,
Runtime:25min,Dilunt:mp

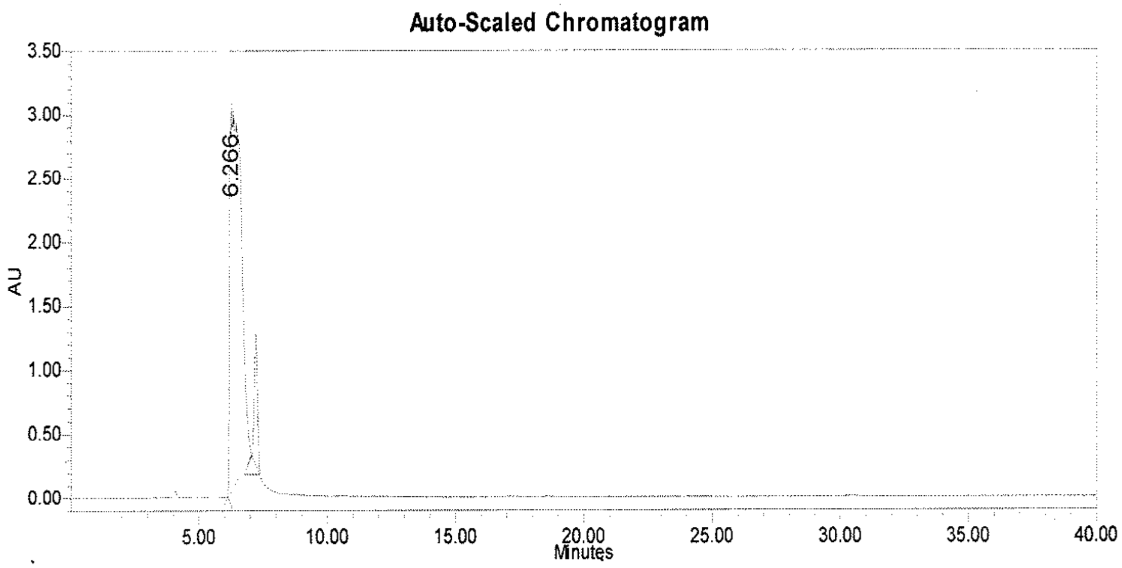

Peak Results

	Name	RT	Area	% Area	Purity1 Angle	Purity1 Threshold	Purity1 Flag	USP Resolution	USP Tailing	% Height	RT Ratio
1		6.266	92918333	100.00					3.344986e+000	100.00	

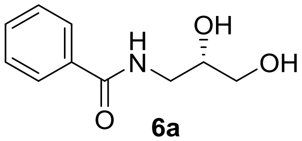

6a

2-RGK-064-.p1d

[Comment]
Sample name
Comment
User swapna
Workgroup ARND
Division
Company DRL

[Data Information]
Creation Date 10/25/2010 11:03 AM

[Measurement Information]
Instrument Name PL/118/002
Model Name P-2000
Serial No. A035861232
Polarizer Glan-Taylor Prism
Faraday Cell Flint Glass

Accessory PTC-203
Accessory S/N A014261234
Path Length 100 mm

Light Source Na
Monitor wavelength 589 nm
D.I.T. 5 sec
No. of cycle 5
Cycle interval 0 sec
Temp. Monitor Cell
Temp. Corr. Factor None
Aperture(S) 3.0mm
Aperture(L) Auto
Mode Specific O.R.
Path Length 100 mm
Concentration 0.759 w/v
Factor 1

		No.	Mode	Calc. Data	Meas. Data	Monitor(deg)	Temperature(C)	Blank
1	*	1	Specific O.R.	-12.4401	-0.0944	-0.0869	23.88	0.0075
2	*	2	Specific O.R.	-12.4532	-0.0945	-0.0870	23.96	0.0075
3	*	3	Specific O.R.	-12.2688	-0.0931	-0.0856	24.02	0.0075
4	*	4	Specific O.R.	-12.4928	-0.0948	-0.0873	24.07	0.0075
5	*	5	Specific O.R.	-12.5455	-0.0952	-0.0877	24.11	0.0075
6	*	6		-12.4401				
7		7		0.1042				
8		8		0.8373				

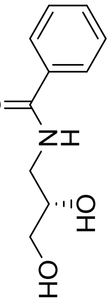

6a

Elemental Composition Report

Page 1

Single Mass Analysis

Tolerance = 5.0 PPM / DBE: min = -1.5, max = 80.0
Element prediction: Off
Number of isotope peaks used for i-FIT = 3

Monoisotopic Mass, Even Electron Ions
97 formula(e) evaluated with 1 results within limits (up to 4 best isotopic matches for each mass)
Elements Used:
C: 0-30 H: 0-30 N: 0-5 O: 0-6
3/RGK/084
UT1211_033 22 (0.413) Cm (21:25)

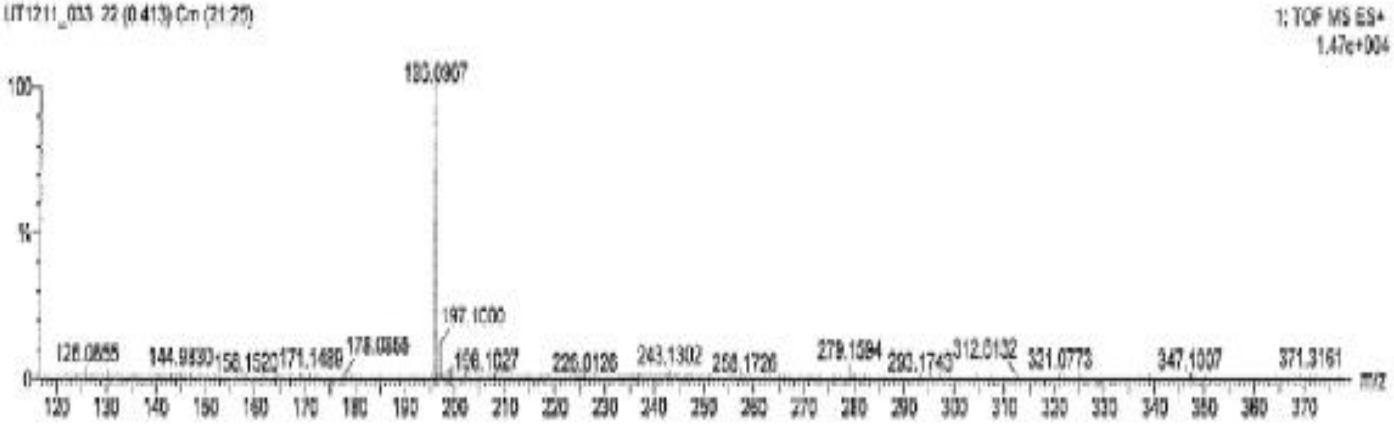

Minimum:				-1.5		
Maximum:		5.0	5.0	80.0		
Mass	Calc. Mass	mDa	PPM	DBE	i-FIT	Formula
196.0967	196.0974	-0.7	-3.6	4.5	2.5	C10 H14 N O3

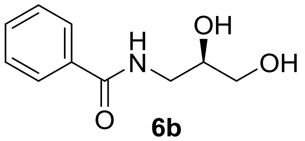

6b

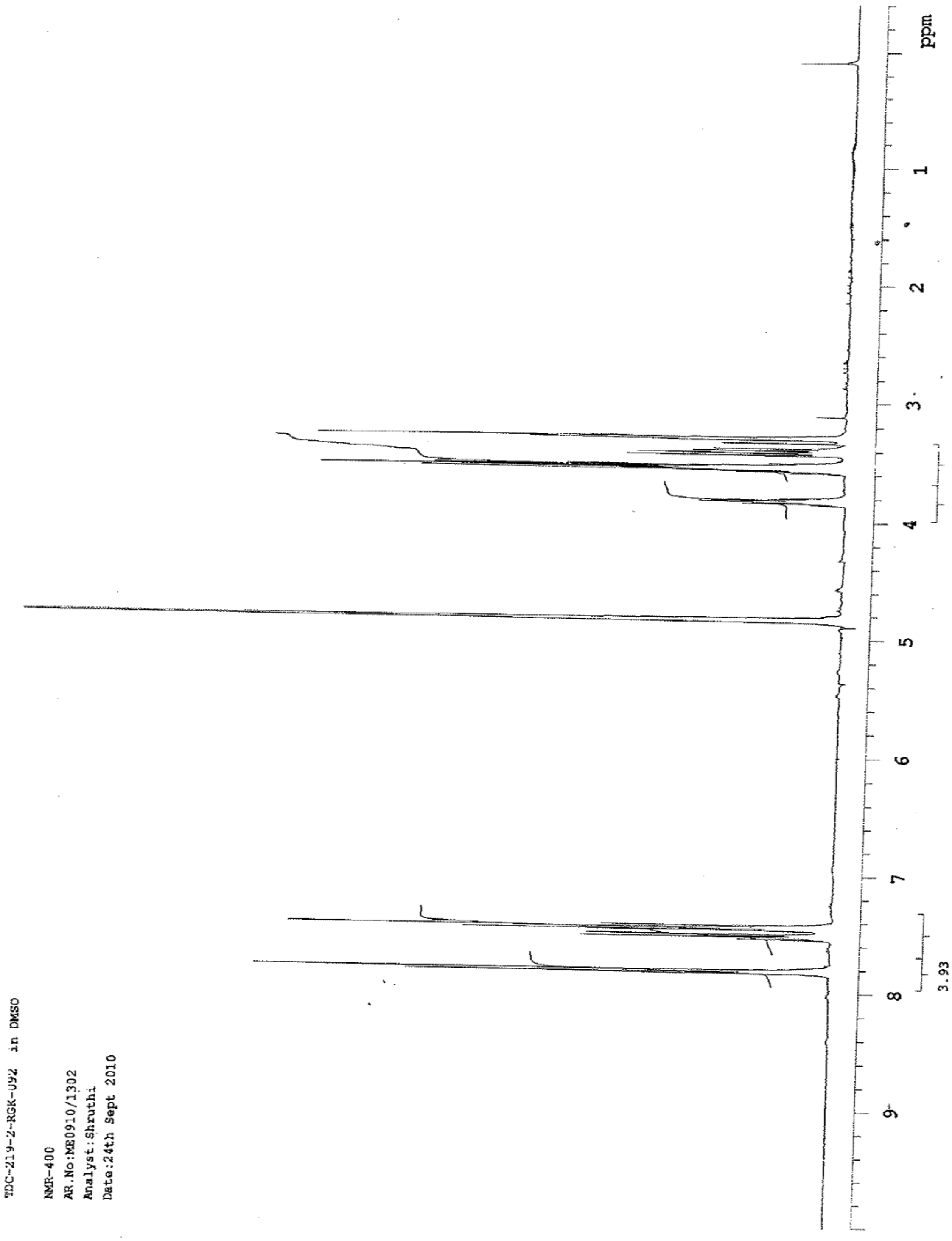
TDC-219-2-RGK-092 in DMSO
NMR-400
AR.No:ME0910/1302
Analyst:Shruthi
Date:24th Sept 2010
ppm
1
2
3
4
5
6
7
8
9
2.00
8.32
3.93
5.72

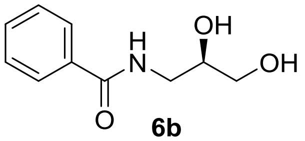

6b

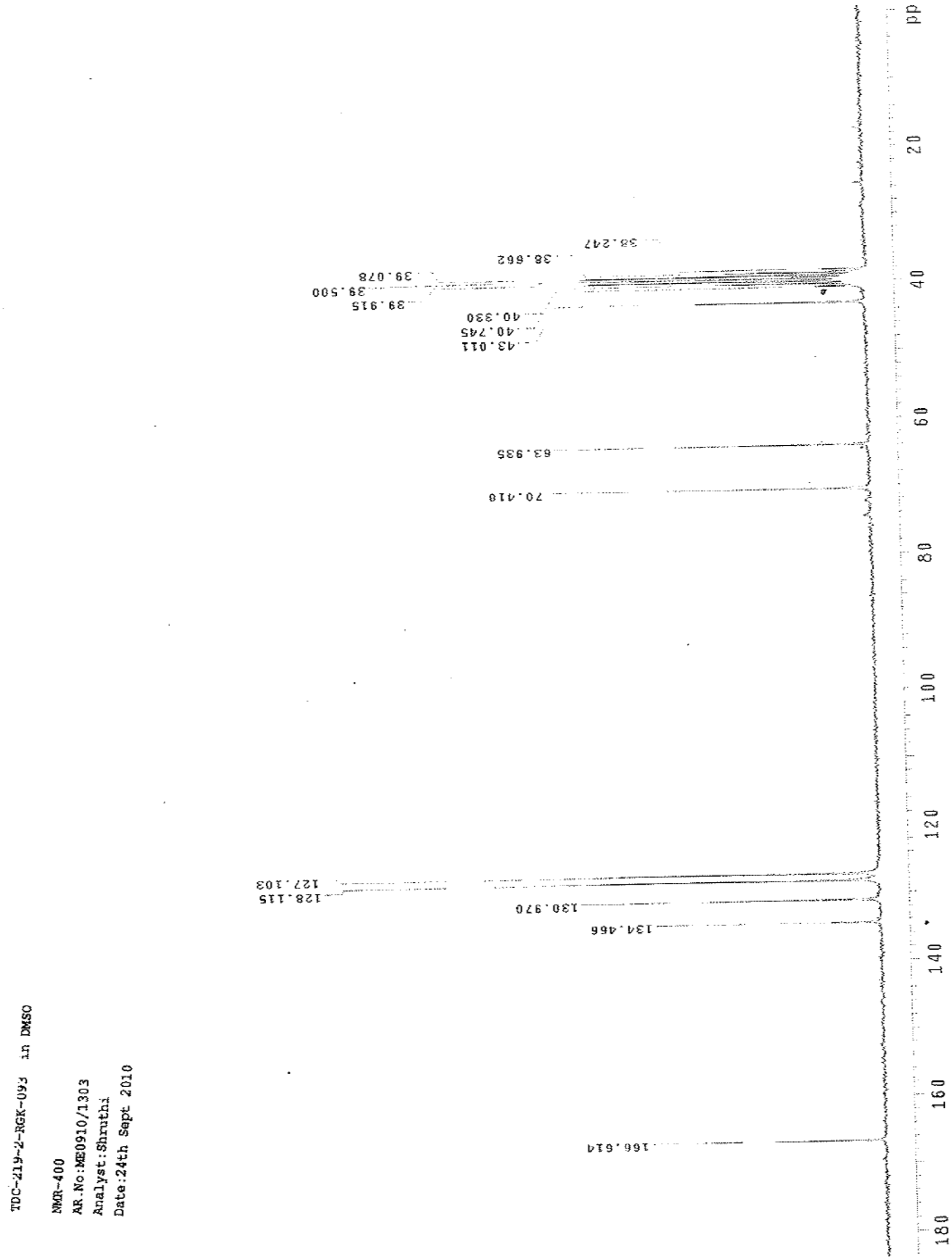
TDC-219-2-RGK-093 in DMSO
NMR-400
AR.No:ME0910/1303
Analyst:Shruthi
Date:24th Sept 2010
38.247
38.662
39.078
39.500
39.915
40.330
40.745
43.011
63.935
70.410
127.103
128.115
130.970
134.466
166.614
ppm
20
40
60
80
100
120
140
160
180

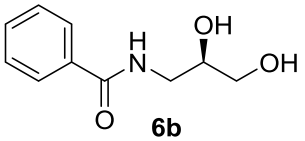

6b

SAMPLE INFORMATION

Sample Name:	2/RGK/092	Acquired By:	System
Sample Type:	Unknown	Date Acquired:	10/1/2010 11:59:23 AM
Vial:	25	Acq. Method Set:	CIRA2
Injection #:	1	Date Processed:	10/11/2010 5:10:43 PM
Injection Volume:	10.00 ul	Processing Methoc	cira2
Run Time:	40.0 Minutes	Channel Name:	Wvln Ch9
Sample Set Name	10100101	Proc. Chnl. Descr.	PDA 220.0 nm

Coloumn: Chiral pak IA(250x4.6mm,5μ)
Mobile Phase:N-HEXANE:IPA(8:2)
Flow:1.0ml/min,Wave Length:220nm,
Column Temperature:40°C,Inj Vol:10ul,
Runtime:25min,Dilunt:mp

Auto-Scaled Chromatogram

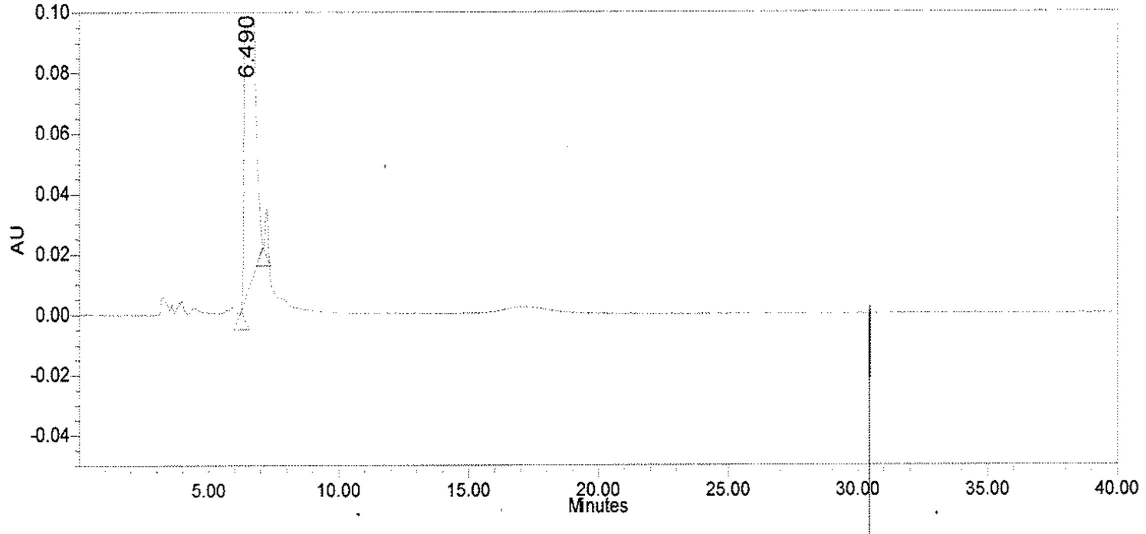

Peak Results

	Name	RT	Area	% Area	Purity1 Angle	Purity1 Threshold	Purity1 Flag	USP Resolution	USP Tailing	% Height	RT Ratio
1		6.490	5921473	100.00					1.790058e+000	100.00	

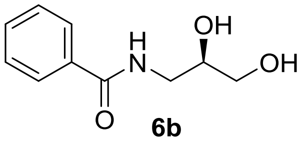

6b

2-RGK-092 - RIsomer.p1d

[Comment]
Sample name
Comment
User ramanjaneyulu
Workgroup ARND
Division
Company DRL

[Data Information]
Creation Date 10/19/2010 3:34 PM

[Measurement Information]
Instrument Name PL/118/002
Model Name P-2000
Serial No. A035861232
Polarizer Glan-Taylor Prism
Faraday Cell Flint Glass

Accessory PTC-203
Accessory S/N A014261234
Path Length 100 mm

Light Source Na
Monitor wavelength 589 nm
D.I.T. 5 sec
No. of cycle 5
Cycle interval 0 sec
Temp. Monitor Cell
Temp. Corr. Factor None
Aperture(S) 3.0mm
Aperture(L) Auto
Mode Specific O.R.
Path Length 100 mm
Concentration 0.88 w/v
Factor 1

		No.	Mode	Calc. Data	Meas. Data	Monitor(deg)	Temperature(C)	Blank
1	*	1	Specific O.R.	12.3341	0.1085	0.1085	23.40	-0.0000
2	*	2	Specific O.R.	11.9818	0.1054	0.1054	23.47	-0.0000
3	*	3	Specific O.R.	12.0841	0.1063	0.1063	23.51	-0.0000
4	*	4	Specific O.R.	12.3341	0.1085	0.1085	23.54	-0.0000
5	*	5	Specific O.R.	12.2432	0.1077	0.1077	23.56	-0.0000
6	*	6		12.1955				
7		7		0.1571				
8		8		1.2884				

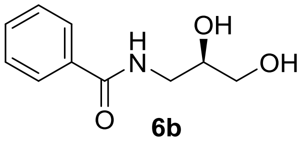

OH
H
N
OH
O

6b

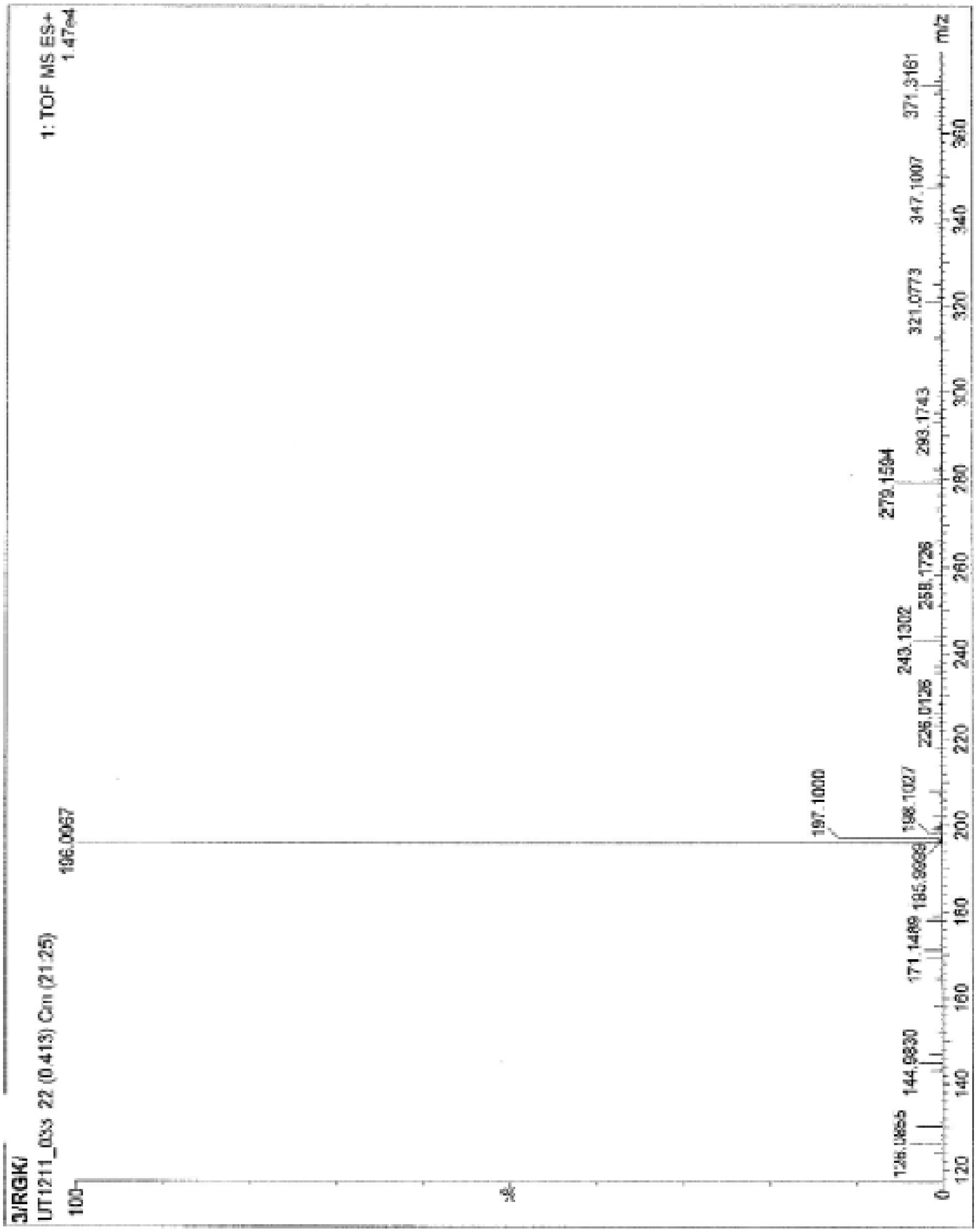

3/RGK/
UT1211_033 22 (0.413) Cm (21:25)
1: TOF MS ES+
1.47e4
196.0967
197.1000
198.1007
279.1594
m/z
